# Unraveling the Role of the Multifunctional Groups in the Adsorption of l‑Cysteine on Rutile TiO_2_(110)

**DOI:** 10.1021/jacs.5c07119

**Published:** 2025-10-22

**Authors:** Miguel Blanco Garcia, Daniele Perilli, Chiara Daldossi, Aldo Ugolotti, Martina Giordano, Daniel Silvan Dolling, Michael Wagstaffe, Mona Kohantorabi, Andreas Stierle, Cristiana Di Valentin, Heshmat Noei

**Affiliations:** † Centre for X-Ray and Nano Science CXNS, 28332 Deutsches Elektronen-Synchrotron DESY, Hamburg 22603, Germany; ‡ University of Hamburg, Notkestraße 9-11, Hamburg 22607, Germany; § Department of Materials Science, 9305 University of Milano-Bicocca, Via R. Cozzi 55, Milano I-20125, Italy; ∥ BioNanoMedicine Center NANOMIB, University of Milano-Bicocca, Milano I-20125, Italy

## Abstract

Understanding the interaction between biomolecules and oxide surfaces is essential for advancing technologies in photocatalysis, virus inactivation, and self-cleaning materials. This study investigates the adsorption behavior of l-cysteine on the rutile TiO_2_(110) surface using a combined experimental and theoretical approach. By employing X-ray photoelectron spectroscopy (XPS), Fourier-transform infrared reflection absorption spectroscopy (FT-IRRAS), scanning tunneling microscopy (STM), and density functional theory (DFT) calculations, we elucidate the molecular configurations and bonding mechanisms involved in the interaction of cysteine with the TiO_2_ surface. The results reveal three distinct adsorption geometries: two bidentate bridging modes involving the carboxylate group and amino group and a configuration involving the interaction of the thiolate group with titanium atoms. Additionally, cysteine molecules form dimers stabilized by disulfide bonds even at low coverage while maintaining a zwitterionic state. Our study highlights, for the first time, the key role of the thiol group in cysteine adsorption on TiO_2_, both for surface direct binding and dimer formation. These findings provide new insights into the fundamental principles of biomolecule–semiconductor interactions with important implications for surface-functionalized materials in catalysis and sensing.

## Graphical abstract

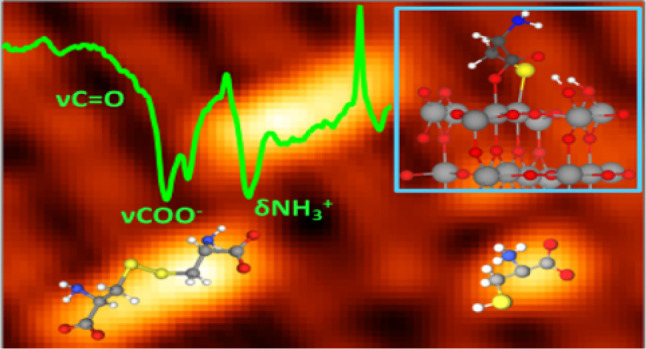

## Introduction

Titanium dioxide (TiO_2_) is renowned for its remarkable photocatalytic properties, enabling critical chemical processes such as water splitting,^1^ degrading organic pollutants,^2^ and microorganisms.^3^ These properties, along with the fact that TiO_2_ is a nontoxic compound and is widely available, have made it central to various industrial applications, including water treatment,^4^ disinfection technologies, such as antiviral, self-cleaning surfaces,^5,6^ and air purification systems.^7^

TiO_2_ has demonstrated excellent efficacy in inactivating viruses. Recent studies have highlighted its ability to neutralize the severe acute respiratory syndrome coronavirus 2 (SARS-CoV-2) by targeting and degrading the virus’s outermost layer (spike proteins).^8,9^ Previous investigations have demonstrated that degradation of the spike proteins can lead to the ultimate inactivation of the virus, increasing the interest in understanding the interaction between the spike proteins at the interface with the TiO_2_ surfaces.^10,11^

The rutile (110) surface is the thermodynamically most stable termination of rutile and, moreover, is one of the most extensively studied oxide surfaces.^12,13^ Numerous studies have explored the adsorption behavior of various small and large molecules on the TiO_2_(110) surface, including water,^14^ carboxylic acids,^15–17^ and lipids among others.^18–27^ Understanding its interaction with amino acids, the protein building blocks, is a crucial step to fully understand the interaction behavior of the virus and other pathogens with the surface of oxide photocatalysts.

Several experimental and computational studies have investigated the adsorption of amino acids on rutile TiO_2_(110).^28,29^ While previous findings suggest a preferred adsorption mode via dissociation of the carboxylic group, variations in behavior arise depending on the type of the amino acid and the presence of water at the surface. For instance, Yazdanyar et al. found that the backbone of amino acids, comprising the amine and carboxyl groups, universally participates in adsorption across all types of amino acids while only the polar or charged side-chains can promote the adsorption process.^30^

Among amino acids, l-cysteine (cys) stands out for its unique characteristics. As one of the smallest amino acids, it exhibits significant chemical complexity due to the presence of three distinct functional groups: carbonyl, amino, and thiol. Notably, l-cysteine is the only amino acid containing a thiol group. Additionally, the tendency of cysteine molecules to form disulfide bonds is well-known for strengthening the structural integrity and three-dimensional stability of proteins.^31,32^ Also, cysteine is one of the most abundant amino acids in the spike protein of SARS-CoV-2, highlighting the importance of understanding the adsorption behavior of this molecule with photoactive catalysts.^33^

Despite experimental^34,35^ and theoretical^36,37^ investigations, the precise nature of the cysteine-TiO_2_ interactions remains unresolved due to inconsistencies between observed and predicted results. Pantaleone et al. proposed, based on DFT conclusions, that cysteine preferentially adsorbs on the TiO_2_(110) surface in a bidentate bridging configuration (O,O), where the carboxylate group interacts with two 5-fold-coordinated Ti atoms (Ti_5c_).^36^ The authors suggested that both deprotonated and zwitterionic cysteine configurations are energetically similar, with the sulfur group remaining unbound to the surface. Muir and Idriss computed two stable binding modes: the same bridging bidentate COO-Ti (O,O) and also a bridging through Ti–OOC-H_2_N–Ti (O)-(N), through DFT calculations.^37^ In some of the modes in which cysteine is binding through (O,O), the authors observed a stabilization interaction through the thiol group via proton transfer with a surface oxygen. The involvement of the thiol group in cysteine adsorption was also suggested by classical nonreactive and reactive force field calculations.^38,39^ An experimental study using XPS^34^ reported the cysteine adsorption through the carboxylate group in a bridging bidentate (O,O) configuration with sulfur atoms interacting with titanium ions at oxygen vacancy sites and the subsequent cleavage of the sulfur–carbon bond. However, these experimental findings diverge from all theoretical models, which have consistently predicted a stable cysteine adsorption on the rutile (110) surface without any sulfur–carbon bond rupture.

Consequently, several critical questions remain unanswered regarding the specific adsorption configurations of cysteine (molecular, dissociated, or zwitterionic), the precise bonding nature and location of bridging interactions ((O,O) or (O)-(N)), the role of the thiol group in adsorption, the specific adsorption site on the TiO_2_ surface, and potential intermolecular interactions between adsorbed cysteine molecules. Addressing these knowledge gaps is essential for gaining a deeper understanding of the cysteine-TiO_2_ interface and increasing the feasibility of TiO_2_ as a self-cleaning material and virus inactivator.

To comprehensively investigate the complex cysteine-TiO_2_(110) interface, we utilize a controlled environment using ultrahigh vacuum (UHV) conditions combined with a multitechnique approach. XPS and FT-IRRAS were utilized to identify the specific chemical bonds and geometries of the adsorbed features involved in the cysteine-TiO_2_ interaction. Additionally, STM provided further insights into the adsorption morphology and arrangement of cysteine molecules on the rutile surface. To complement the experimental findings and gain a deeper understanding of the bond-making and the role of multifunctional groups in the cysteine-TiO_2_ system, theoretical calculations were performed based on DFT. Our results reveal, for the first time, the presence of three distinct adsorption configurations of cysteine on rutile TiO_2_(110) at room temperature. Furthermore, we observed the presence of cysteine dimers at low coverages and a preference for the zwitterionic configuration at higher cystine coverages on the TiO_2_(110) surface.

## Results and Discussion

Cysteine molecules are known to adsorb in multiple configurations on the rutile (110) surface. In this work, we systematically investigate the most relevant configurations, combining an experimental and theoretical approach to gain deeper insights into the adsorption mechanisms.

### Nomenclature for Cysteine Adsorption on TiO_2_

We have investigated the cysteine adsorption on the rutile surface in different protonation states, as schematically shown in Scheme 1a: *molecular* (indicated with M), with its carboxylic, amino, and thiol groups in their canonical form; *deprotonated* (or DP), where the carboxylic group loses the hydrogen forming a carboxylate COO^–^; *zwitterionic* (or ZW), where the dissociated hydrogen from the carboxylic group protonates the amino group to NH_3_^+^; and *bideprotonated* (or biDP), where both the carboxylic and thiol groups are deprotonated.

**Scheme 1:**
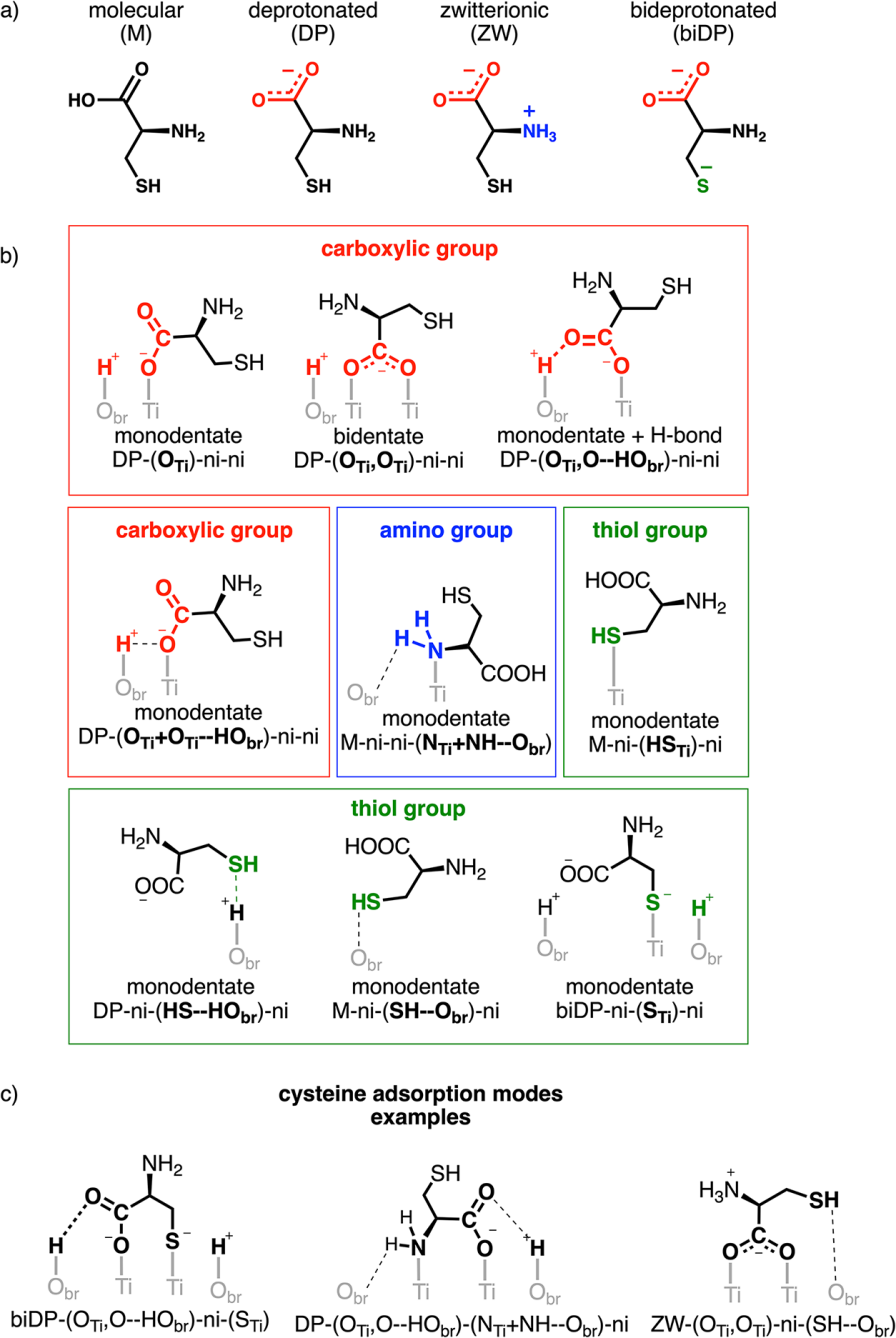
Structures of (a) Investigated Protonation Forms of Cysteine, (b) Possible Adsorption Models of the Different Carboxylic (Red), Amino (Blue), and Thiol (Green) Functional Groups of Cysteine on the TiO_2_ Surface, and (c) Examples of Relevant Cysteine Adsorption Modes

Each cysteine adsorption geometry will be identified by a label made of four blocks separated by a dashed line (see examples in Scheme 1c). The first block indicates the protonation state of the amino acid, followed by three more blocks enclosed by parentheses describing the binding modes of the three functional groups present in cysteine: carboxylate, amino, and thiol, respectively (Scheme 1b).

In particular, in the second block, we indicate in parentheses the atoms of the carboxylate group establishing an interaction with the rutile surface (red section in Scheme 1b) either as a coordinative bond with Ti_5c_ or as an H-bond with surface HO_br_. When both of the O atoms bind to Ti_5c_, this block is (O_Ti_,O_Ti_). If H-bonds are established, they will be named as O–HO_br_.

The third block describes the amino group binding mode to the rutile surface (blue section in Scheme 1b). The NH_2_ group can bind to a surface Ti_5c_ through the N atom (N_Ti_) or form additional H-bonds with the undercoordinated O_br_ sites (NH–HO_br_). When both of these happen, this block is (N_Ti_+NH–HO_br_).

Finally, the fourth block defines the thiol group binding mode to the rutile surface (green section in Scheme 1b). The SH group can bind to a surface Ti_5c_ through the S atom (S_Ti_) before or after dissociation of the S–H bond, or it can establish electrostatic interactions with the undercoordinated O_br_ sites (SH–O_br_ or HS–HO_br_).

When a functional group is not interacting with the TiO_2_ surface, the nomenclature “ni” (noninteracting) will be used without parentheses.

### Deprotonated Cysteine

We start by presenting the possible adsorption modes of isolated cysteine on a rutile (110) surface slab model that we have obtained as stable optimized structures by means of full atomic relaxation with DFT methods. The computational details can be consulted in the Supporting Information. Due to the inclusion of Hubbard U and Grimme corrections,^40–43^ our results are not in complete agreement with a previous study by Muir and Idriss as it will be discussed below.^37^

Our calculations indicate that the carboxylic group of cysteine dissociates upon adsorption (deprotonated cysteine, DP), transferring a proton to a nearby surface bridging O atom. This process involves one or two under-coordinated surface Ti_5c_ atoms, which form coordinative bonds with one or two carboxylate O atoms (monodentate or bidentate), as shown in Scheme 1. In all cases that we investigated, the deprotonated cysteine molecules establish at least two or more different interactions with the surface atoms. The most stable geometries, those having the lowest (i.e., most negative) adsorption energies, are illustrated in Figures 1 and S3. In the first configuration (Figure 1a), two interactions are the bidentate binding of the two carboxylate O with two neighboring Ti_5c_, whereas the third is an electrostatic attraction between the S atom of the thiol group with the dissociated proton bound to a surface bridging O atom (DP-(O_Ti_,O_Ti_)-ni-(HS–HO_br_)). In the second configuration (Figure 1b), one interaction is the coordinative bond of one carboxylate group O with a surface Ti_5c_ atom, the second interaction is the coordinative bond of the N atom of the amino group with another surface Ti_5c_ atom, and then there are two H-bonds established between the bridging OH group with the unbound carboxylate O atom and between the NH group of amino with a surface bridging O atom (DP-(O_Ti_,O–HO_br_)-(N_Ti_+NH–O_br_)-ni). We also explored the involvement of the thiol group in the cysteine surface adsorption, as suggested by Monti et al.,^38,39^ and reported the most stable structures. In the third configuration (Figure 1c), one interaction is the coordinative bond of one carboxylate O with a surface Ti_5c_ atom, and the second interaction is the coordinative bond of the S atom of the dissociated thiol group with another surface Ti_5c_ atom (biDP-(O_Ti_,O–HO_br_)-ni-(S_Ti_)). Additionally, in the biDP-(O_Ti_,O–HO_br_)-ni-(S_Ti_) structure, one H-bond is established between one bridging surface OH group with the unbound carboxylate O atom, and an electrostatic interaction is formed between the other bridging surface OH group and the S atom. Notably, the biDP-(O_Ti_,O–HO_br_)-(ni)-(S_Ti_) is a highly stable new adsorption configuration that was never reported in previous studies.^36–39^ In the fourth configuration (Figure 1d), one interaction is the coordinative bond of one carboxylate O with a surface Ti_5c_ atom, whereas the second is between the S of the undissociated thiol group and another surface Ti_5c_ atom, although the Ti-SH distance is extremely long (4.53 Å) (DP-(O_Ti_)-ni-(HS_Ti_)).

**Figure 1:**
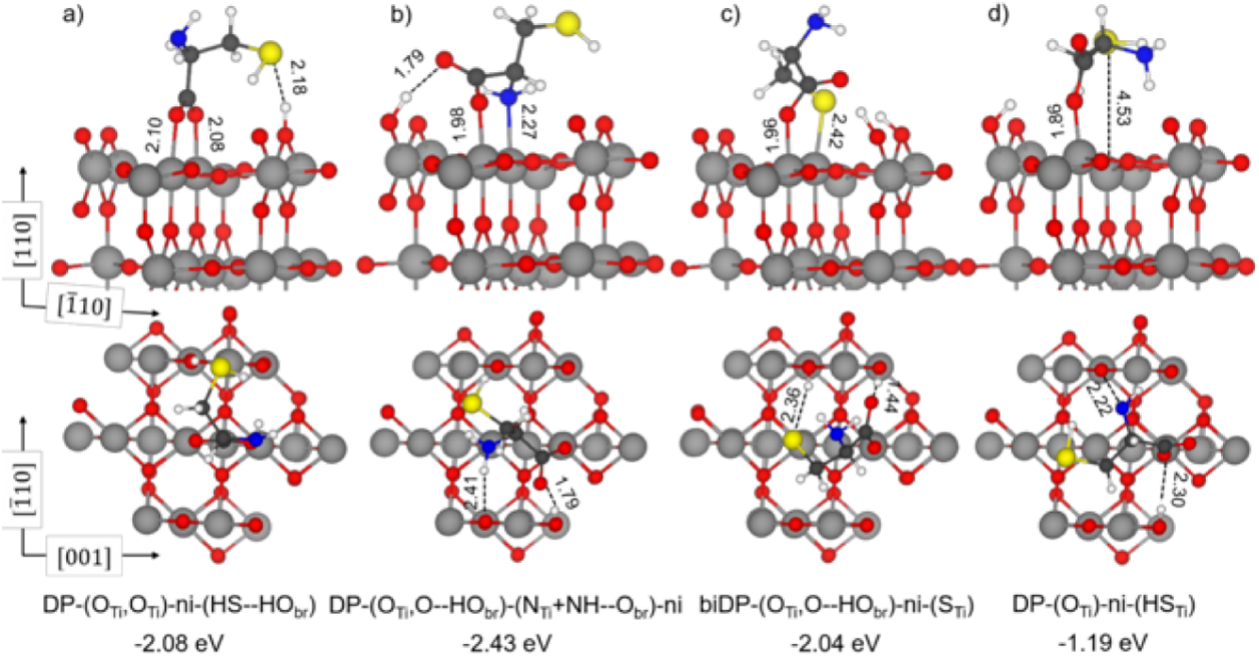
(a-d) Optimized structures of deprotonated cysteine adsorbed on the rutile TiO_2_(110) surface. Gray, red, blue, yellow, white, and black spheres represent Ti, O, N, S, H, and C atoms, respectively. Dashed lines indicate hydrogen bonds and other electrostatic interactions. Values of adsorption energy (in eV) calculated with PBE-D3+U and relevant bond lengths (in Å) are reported.

The trend in the adsorption energy for these four configurations does not change whether the Hubbard U correction is used (Table S1). Therefore, we can conclude that adsorption modes involving direct interaction of the deprotonated thiol S atoms with surface Ti atoms are comparable with those involving carboxylate O atoms. The preferred configuration, however, uses both one carboxylate atom and the amino N atom to coordinate with two surface Ti_5c_. We have also investigated the role played at higher coverages by introducing two cysteine molecules into the same supercell slab model used before (Figure S4). We observed that the structures exhibit the same energy trend as when the molecules were individual adsorbates, but with a slightly enhanced stabilization due to an additional H-bond formed between the two structures (Table S3).

From the experimental side, XPS were obtained on a clean rutile (110) surface before and after cysteine adsorption. The surface preparation, characterization methods, and coverage calculation can be consulted in the Supporting Information. Experiments were performed at the DESY Nanolab.^44^ The clean sample showed no contamination from carbon, hydroxyl, or water in XPS and displayed a LEED pattern representative of a stoichiometric (1 × 1) rutile (110) surface (Figure S1). Cysteine was dosed onto the surface until saturation corresponding to approximately one monolayer was achieved. After cysteine adsorption, the deconvoluted C 1s core-level spectrum, depicted in Figure 2a, reveals three distinct peaks with three chemically inequivalent carbon atoms in a ratio of 1:1:1. The peak with the higher binding energy corresponds to the carboxylic carbon at 289.0 eV. The second peak, at slightly lower binding energies at 286.1 eV, corresponds to the α-carbon of the amino acid. The third peak at 285.2 eV is attributed to the carbon bonded to sulfur. In order to provide an additional tool for comparing the optimized adsorption configurations with the experimental measurements, we performed a spectroscopic characterization through simulated photoemission spectra. However, we cannot calculate absolute values for the binding energies through our approach (see the Supporting Information for further details), but we can only obtain relative differences, i.e., core-level shifts (CLSs). From the calculated CLSs, which are collected in Table S4, it is possible to reveal three spectroscopic features at the C K-edge, separated by 0.6 eV (C–S to C–N) and 1.9 eV (C–N to C–O). Experimentally, these energy differences are 0.9 and 2.9 eV, respectively. Although the calculated energy separations are underestimated compared to experimental results, such discrepancies have been reported previously for other molecules adsorbed on rutile (110) surfaces.^45^ Due to the small energy differences among the computed CLS values for the different geometries, it remains challenging to conclusively assign a specific adsorption geometry only based on the C 1s spectrum. This is a consequence of the presence of a carboxylate group interacting with the surface in all of the adsorption models. In the case of the (O)-(S) geometry, the additional interaction of the deprotonated thiol group with a Ti atom reduces the binding energy of the α-carbon and the C in the thiol side-chain. This observation aligns with previous findings of the adsorption of cysteine on the rutile (110) surface.^34,35,46^ However, contrary to Ataman et al.,^34,35^ we do not observe any degradation or breaking of the cysteine molecule. We believe that the bond breaking observed in their work may be attributed to radiation damage coming from the synchrotron source or contaminants.

**Figure 2:**
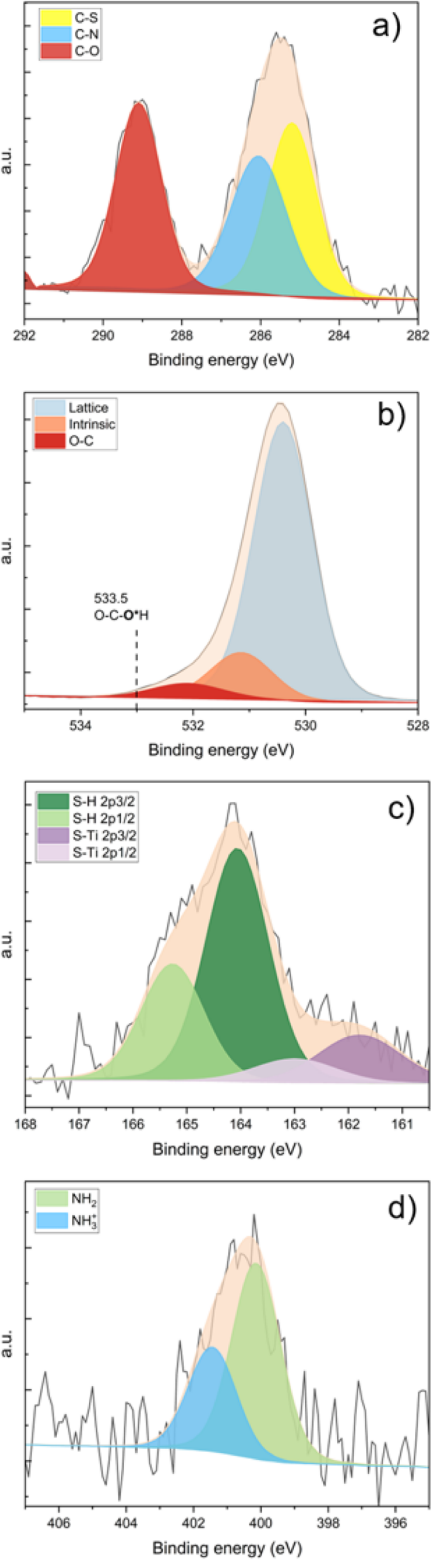
Experimental (black lines) and deconvoluted XP spectra (colored areas) of the (a) C 1s, (b) O 1s, (c) S 2p, and (d) N 1s core levels.

Figure 2b shows the deconvoluted O 1s spectrum for the clean rutile (110) surface. The main peak at 530.4 eV corresponds to lattice oxygen in TiO_2_, while the secondary component at 531.2 eV has been previously linked to either bridging oxygens or hydroxyl species. However, its origin remains debated and may reflect an intrinsic feature of the TiO_2_ lattice or a slight peak asymmetry.^47,48^ Upon cysteine deposition, a new oxygen peak emerged at a binding energy of 532.1 eV. This peak is characteristic of oxygen atoms in carboxylic compounds, as previously observed during glutamic acid adsorption.^49^ Notably, the cysteine molecule contains two distinct oxygen atoms (CO^1^O^2^H), which, if protonated, would typically result in two separate peaks with a binding energy difference of approximately 2 eV, according to our gas-phase calculations. The proton-bonded oxygen would be expected to display a higher binding energy, exceeding 533.5 eV.^50,51^ The absence of this peak implies the loss of an acidic proton upon adsorption on the TiO_2_ surface.

Theoretical calculations provide further insight into the observed XP spectral features. For bidentate (O)-(N) configurations, the two oxygen atoms in cysteine are chemically inequivalent with calculated CLSs that differ by approximately 0.1 eV (see Table S4 for a comparison of the calculated values for the different geometries). Such differences are notably smaller than those calculated for adsorption structures obtained with more approximate ReaxFF simulations.^38,39^ The differences are so small that due to limitations with the achievable experimental resolution, resolving this component is difficult, particularly given the relatively low intensity of this peak compared to the lattice oxygen peak.^52^ For comparison, studies on Fe_3_O_4_ have shown that carboxylate species yield a single peak at 531.6 eV.^53^ Thus, the O 1s spectrum alone cannot definitively distinguish between the bridging (O,O) and (O)-(N) configurations.

Figure 2c illustrates the deconvoluted XP spectra of the S 2p core level, revealing the presence of two distinct sulfur species with the characteristic doublets separated by 2.4 eV. The lower binding energy 2p_3/2_ peak at 161.7 eV is attributed to sulfur bound to the titanium, Ti_5c_, like in structure DP (O)-(S) (Figure 1c), while the higher binding energy 2p_3/2_ peak at 164.1 eV corresponds to the thiol (S–H) group in the cysteine molecule. The different CLSs for S–H in both DP (O)-(N) and DP (O)-(O) are not so big as to resolve with precision these two structures. To adequately fit the spectra, a spin–orbit splitting of 1.2 eV was applied to the S 2p_1/2_ and S 2p_3/2_ peaks, with an area ratio of 1:2. The area ratio between the two sulfur species is 1 (S–H) to 0.3 (S–Ti). Ataman et al. assigned the lower binding energy peak to a sulfur bounded to a titanium atom in an oxygen vacancy.^34,35^ However, our Ti 2p spectra before and after cysteine deposition show no significant change in the Ti^3+^ signal (see Figure S2), maintaining a Ti^4+^/Ti^3+^ ratio of approximately 1:0.05. This low concentration of oxygen vacancies suggests that most cysteine molecules do not interact with defect sites. Instead, the presence of both S–H and S–Ti species likely reflects the coexistence of multiple adsorption mechanisms on the surface at this coverage. Our theoretical simulations verify this hypothesis, as we find two main sulfur features among the different calculated CLSs (see Table S4), corresponding to S–Ti or S–H bonding configurations. These two features are separated by 1.9 eV, which agrees with previous ReaxFF-driven calculations^38,39^ and compares well with the experimental observations (2.4 eV between species). To further clarify the binding mode and geometry of cysteine on TiO_2_, FT-IRRAS was employed (see Figure 3). If the bidentate binding mode (O,O) is the predominant mechanism of the dissociative adsorption of cysteine on rutile (110), we would expect to observe two distinct bands corresponding to the asymmetric (ν_asym_) and symmetric (ν_sym_) stretching vibrations of OCO. Studies performed on other acids, such as formic acid or l-alanine, show that we can expect the ν_asym_(OCO) and ν_sym_(OCO) bands around 1550 and 1370 cm^–1^ on oxide surfaces, respectively.^54,55^

**Figure 3:**
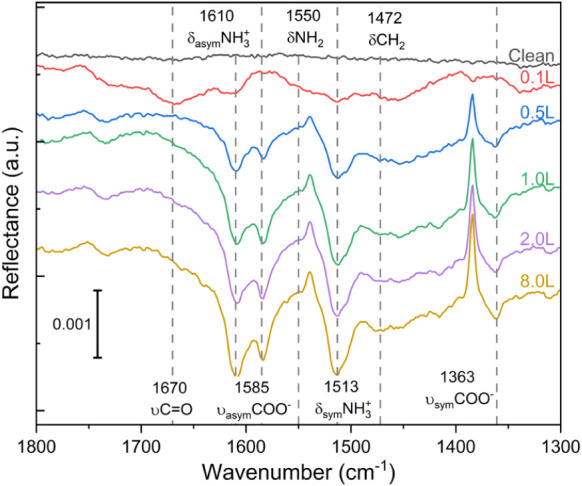
FT-IRRAS spectra for different coverages of cysteine on rutile TiO_2_(110) at room temperature.

At low coverage, the presence of a carbonyl (C=O) stretching vibration is observed at around 1670 cm^–1^. This indicates monodentate adsorption, likely involving either (O)-(N) or (O)-(S). Nara et al.^60^ calculated similar frequencies for C=O stretching configurations where the carboxylic group interacts with a single titanium atom. At higher coverages, additional bands are observed at 1585 and 1363 cm^–1^, assigned to the ν_asym_(OCO) and ν_sym_(OCO), respectively. Pászti and Guczi^61^ describe band positions and splitting for different OCO configurations of adsorbed amino acids on TiO_2_. In monodentate configurations, ν_asym_(O = C–O–Ti) is always above 1600 cm^–1^, similar to carbonyl stretching.

In the case of aspartic and glutamic acids, it is typically around 1660–1680 cm^–1^. For bridging bidentate adsorption (O,O), the separation between ν_asym_(OCO) and ν_asym_(OCO) is around 200 cm^–1^ comparable to what we observe. Similar splittings of 200 cm^–1^ were reported previously for formate adsorption on rutile (110) and on other oxide surfaces.^58,59,62^ For comparison, the dissociative adsorption of formic acid on TiO_2_ showed bands at 1555 and 1370 cm^–1^ and on zinc oxide at 1573 and 1374 cm^–1^.^59,62^ The sharp, upward-pointing band at 1385 cm^–1^ is a reproducible interferometer absorption artifact unrelated to cysteine adsorption. The corresponding background trace is provided in Figure S10.

To support the assignment of the experimental peaks, we computed the vibrational normal modes and corresponding frequencies for the three most stable deprotonated configurations (Figure 1a-c). The theoretical frequencies were then compared to the experimental data. The results, summarized in Table 1, show an overall good agreement between theory and experiment. To achieve high accuracy in the vibrational analysis, hybrid DFT methods are considered superior to standard DFT approaches such as GGA-PBE, which was used in the present study (PBE-D3+U).^63^

**Table 1:** Vibration Frequencies Assignment in Figure 3 ^a^

	Experiments		Theory			
Assignment	Wavenumber	Reference	DP-(O_Ti_,O–HO_br_)-(N_Ti_+NH–O_br_)-ni	DP-(O_Ti_,O_Ti_)-ni-(HS–HO_br_)	biDP-(O_Ti_,O–HO_br_)-ni-(S_Ti_)	ZW-(O_Ti_,O_Ti_)-ni-(SH–O_br_) + 1H_2_O
νC=O	1670	^54–59^	1622	–	1526	–
δ_asym_NH_3_^+^	1610	^55–57^	–	–	–	1582
ν_asym_COO^–^	1585	^54–59^	–	1502	–	1553
δNH_2_	1550	^55–57^	1561	1586	1608	–
δ_sym_NH_3_^+^	1513	^55–57^	–	–	–	1431
δCH_2_	1472	^56,57^	1418	1424	1392	1472
ν_sym_COO^–^	1363	^54–59^	–	1395	–	1376

a Computed values are reported for the three most stable deprotonated configurations and the zwitterionic form. Modes that are absent in a given structure are indicated with a dash (−).

We also assessed the thermodynamic stability at room temperature of the most stable adsorption modes (Figure 1a-c) by computing the Gibbs free energies of adsorption (Δ*G*). From these values, we estimated the corresponding desorption temperatures using the Redhead approximation,^64^ assuming a pre-exponential factor of 10^13^ Hz and a heating rate of 1 K/s, as reported in Table S6. All Δ*G* values are sufficiently negative to ensure stable adsorption at RT and up to *T* < 146 °C.

These results, in combination with XPS data and the theoretical models, clearly indicate that cysteine adsorbs on the TiO_2_ surface in three distinct configurations: bidentate (O,O), bidentate (O)-(N), and bidentate (O)-(S) (Figure 1a-c).

### Zwitterionic Cysteine

Figure 2d presents the deconvoluted XP spectrum of the N 1s core level, revealing two distinct nitrogen peaks. The lower binding energy peak, colored green, at 400.1 eV is assigned to the amino (−NH_2_) group. The calculated CLSs for this nonprotonated group are similar for the various structural models and match those predicted for the NH_2_ group of the molecule in the gas phase (see Table S4). Similar differences were reported in the case of adsorption configurations obtained through ReaxFF calculations.^38^ Slightly less negative CLSs are observed in configurations where the amino group interacts more strongly with the surface, with either a Ti_5c_ or an O_br_ atom. This behavior is similar to what was previously calculated for cysteine adsorbed on the anatase TiO_2_(101) surface through the formation of a hydrogen bond.^65^ However, due to the limited spectral resolution and the broadness of the peaks, combined with the small energy separation between the different NH_2_ species, it is not possible to distinguish between both of them experimentally. Therefore, both strongly and weakly interacting NH_2_ species are grouped under a single NH_2_ assignment.

The higher binding energy peak in blue is assigned to (−NH_3_^+^). The observed peak splitting of 1.5 eV is consistent with previous experimental results; a splitting of 1.5 eV has been reported before for glutamic acid on rutile (110) and falls within the 1.5–2.5 eV range commonly reported for amino acids on various surfaces,^66–70^ as well as in cysteine films.^35^

FT-IRRAS measurements further confirm the protonation of the amino group. The asymmetric deformation band of NH_3_^+^ at 1610 cm^– 1^ and the symmetric deformation band at 1513 cm^– 1^ increase in intensity at higher coverage. Tillotson et al.^71^ calculated that glycine adsorbs on rutile (110) with the amino group forming hydrogen bonds, stabilizing NH_3_^+^. Similarly, Pantaleone et al.^36^ found that the zwitterionic form is energetically close to the deprotonated form, even in the absence of water or surface defects. In our spectra, the NH_2_ deformation band appears as a shoulder at 1550 cm^–1^.^72–75^ These findings indicate that cysteine can adsorb on the surface in either a deprotonated or zwitterionic configuration. To gain deeper insights into this behavior, we performed theoretical calculations to evaluate the stability of the zwitterionic form of cysteine. Specifically, we investigated the possibility that isolated cysteine molecules on the rutile (110) surface slab model are adsorbed in a zwitterionic configuration (zwitterionic cysteine, ZW), where the proton from the carboxylic group is transferred to the amino group rather than to the surface. If the amino group becomes protonated, it cannot bind to a surface Ti_5c_ atom; therefore, the most stable configuration of the DP form is not considered. To have a protonated amino group (−NH_3_^+^), the anchoring groups are expected to be the two carboxylate O atoms coordinated to two surface Ti_5c_ atoms in a bidentate fashion, as shown in Figure 4a for ZW-(O_Ti_,O_Ti_)-ni-(SH–O_br_). This configuration was localized as a minimum energy structure; however, our simulations show it to be less stable than the most favored DP (O)-(N) (i.e., DP-(O_Ti_,O-HO_br_)-(N_Ti_+NH–O_br_)-ni in Figure 1b) by +0.51 eV (Table S1). An increase in the stability of the ZW form is expected by some water solvation. Although cysteine molecules are deposited under UHV conditions, the presence of trace amounts of water in the experimental chamber cannot be entirely ruled out. To investigate this possibility, we added first one water molecule to the system, forming one H-bond with the −NH_3_^+^ group as the proton donor (Figure 4b,c); however, no significant relative stabilization for ZW is observed (with an energy difference with respect to the DP of +0.39 eV). Therefore, we decided to add three water molecules (Figure 4d,e) to create the first solvation shell. The stabilization of the zwitterionic cysteine is now far larger than the DP-(O)-(N) structure solvated by three water molecules (in Figure 4c), resulting in an adsorption energy difference between the two of +0.15 eV. Therefore, under slightly “hydrated” conditions, ZW and DP forms become competitive and could simultaneously be present on the surface. Water contamination is a well-studied phenomenon in UHV. Despite careful sample preparation, water can still be introduced onto the surface during the evaporation process using a load lock at 10^–9^ mbar and a gas line at 10^–5^ mbar, as well as from the cysteine powder itself.^76,77^

**Figure 4:**
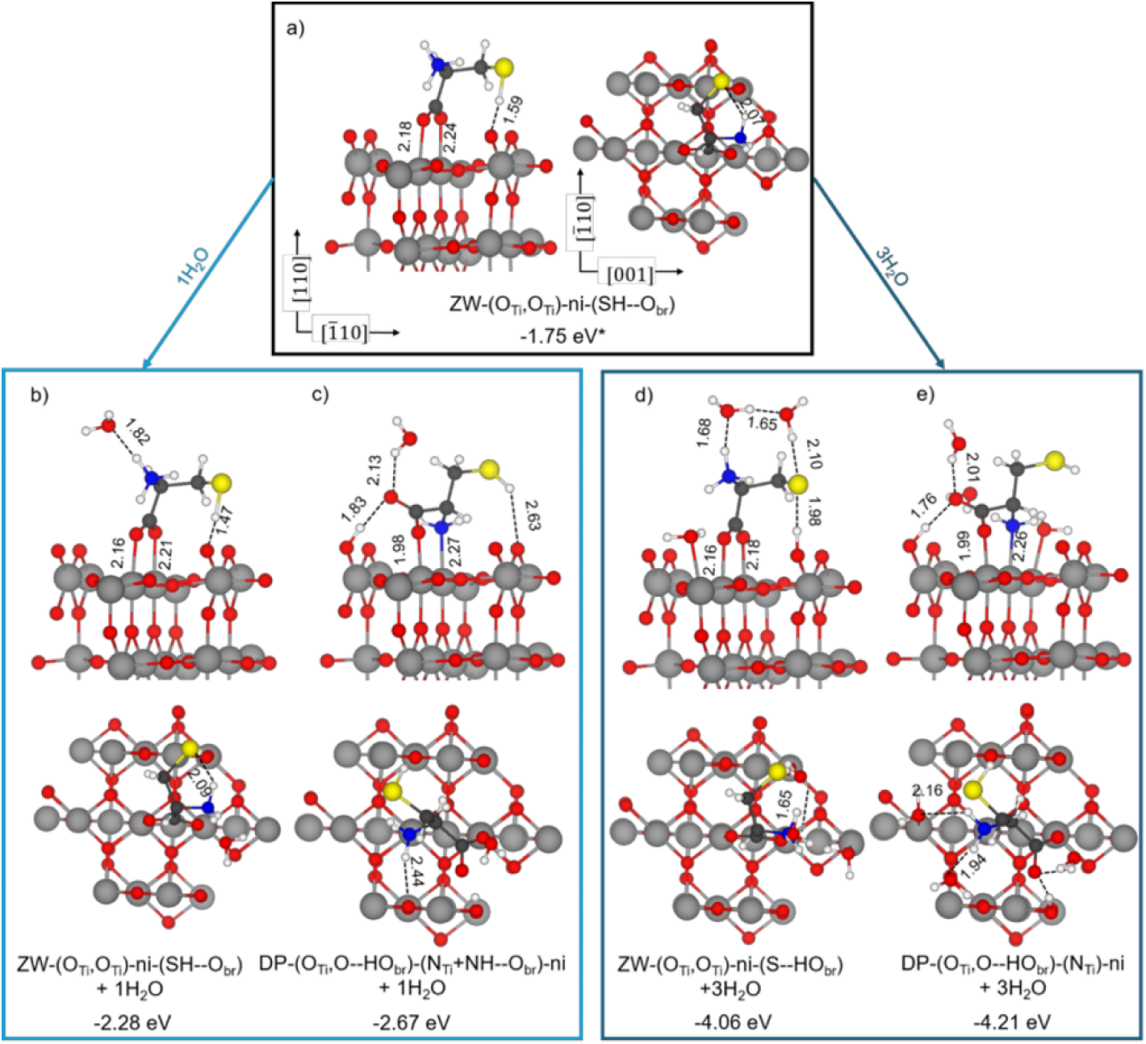
Structures of zwitterionic cysteine adsorbed on the rutile TiO_2_(110) surface in (a) dry and (b,d) “hydrated” conditions, compared with the most stable deprotonated cysteine (c,e) DP-(O_Ti_,O-HO_br_)-(N_Ti_,NH–O_br_)-ni. Gray, red, blue, yellow, white, and black spheres represent Ti, O, N, S, H, and C atoms, respectively. Dashed lines indicate hydrogen bonds and other electrostatic interactions. Adsorption energies (in eV) calculated with PBE-D3+U and relevant bond lengths (values in Å) are reported. (*) For the ZW-(O_Ti_,O_Ti_)-ni-(SH–O_br_), the adsorption energy values are calculated with PBE-D3 without Hubbard U.

A comparison of the XPS characterization of adsorbed zwitterionic cysteine with the deprotonated structures (Table S4) reveals two key differences compared with the DP structures. First, the αC CLS shifts to higher binding energies by approximately 1 eV. Second, and more significantly, a new chemical environment appears, corresponding to the NH_3_^+^ group, with its CLS shifting by as much as 3.5 eV to higher binding energies. This shift is far from the experimental results of 1.5 eV, but this can be explained by the inaccurate screening of the proton charge at the N site, given by our choice to model the polarizing environment through the presence of a few water molecules. However, the CLS we computed for the isolated molecule in the zwitterionic form vs the neutral one is in good agreement with that already reported in previous theoretical studies.^38,39^ Finally, as done for the deprotonated configurations, we also performed a charge analysis and NCI analysis for the zwitterionic configuration with one water molecule (Table S8 and Figure S8). Additionally, we computed the vibrational frequencies of this model structure in Figure 4b, as reported in Table 1. The computed vibrational vectors reproduce both the asymmetric and symmetric bending modes of the NH_3_^+^ group observed experimentally, although one must notice that the associated calculated frequencies are both systematically red-shifted with respect to the experimental bands, likely as a consequence of the use of U-corrected standard DFT instead of a hybrid DFT method and the poor screening of the positive charge in the calculation.

### Cysteine Dimerization

To gain deeper insight into the adsorption geometry and surface morphology of cysteine in TiO_2_, we conducted an STM study. The rutile (110) surface has been extensively explored in numerous STM studies. It exhibits alternating bright rows of 5-fold-coordinated titanium atoms (Ti_5c_) along the [001] direction separated by darker regions corresponding to bridging oxygen rows (O_br_).^12^ Previous STM studies have shown that carboxylic acids preferentially adsorb on top of bright Ti_5c_ rows, typically via bridging configurations involving two adjacent Ti_5c_ atoms. Alternative adsorption sites involving vacancies have also been suggested under certain conditions.^78,79^ Qiu and Barteau^80^ studied glycine adsorption on rutile TiO_2_(110) using STM and found that at higher coverages, glycine forms a (2 × 1) structure, binding to Ti_5c_ atoms. They suggested that the acidic proton migrates to the amino group, forming zwitterionic species, similar to cysteine adsorption. Our spectroscopy results indicate that cysteine adopts a similar bridging geometry, likely in either (O,O) or (O)-(N) configurations.

Figure 5a shows the STM image of the adsorption of 0.1 L of cysteine on rutile (110). This image enables the identification of individual adsorbates, each with an approximate size of 6.4 ± 0.3 Å. In the magnified view (Figure 5c), individual adsorbates are distinctly observed on top of the Ti_5c_ row (bright rows), further supporting a bridging configuration [(O,O), (O)-(N), or (O)-(S)]. These findings strongly support the FT-IRRAS, XPS results, and the DFT calculations.

**Figure 5:**
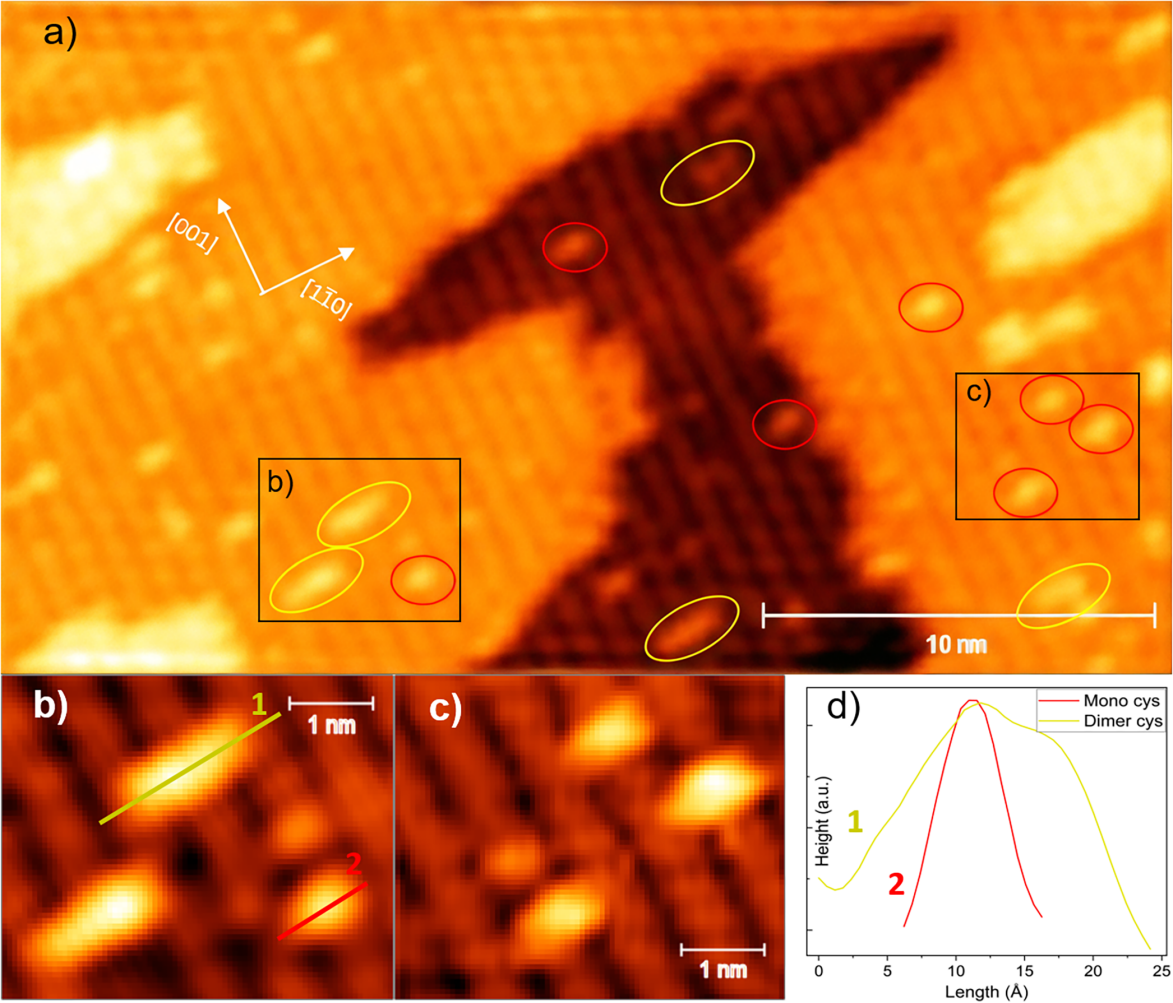
(a) STM image of the rutile (110) surface after dosing 0.1 L of cysteine at room temperature. 30 × 18 nm; image taken with 1.7 V of bias voltage and 0.3 nA current. (b,c) Magnification; (d) dimer and monomer line scans.

The features marked by yellow circles exhibit a total length of 15 ± 1 Å, in which it is possible to distinguish two smaller components each measuring 7.8 ± 0.7 Å. These features likely correspond to cysteine dimers, with each smaller unit slightly exceeding the size of an individual cysteine molecule. Across all STM images acquired under the same conditions, dimers are observed with an approximate frequency of one for every four individual adsorbates (see Figure S9). This dimerization behavior has been previously observed for larger organic molecules on rutile (110) at low coverages. For instance, Schnadt et al.^81^ found that although isonicotinic acid is able to adsorb on rutile (110) as a stable monomer, it was possible to observe dimers and even tetramers of these molecules in STM images. They attributed this tendency of dimerization to a ring interaction. The formation of amino acid dimers on different surfaces is also well-documented in the literature. A review article^82^ describes the dimerization process across various amino acids, such as serine^83,84^ and methionine,^85,86^ on different metal substrates like single-crystalline copper, gold, and silver surfaces.

Cysteine’s ability to form dimers on different surfaces has been extensively studied both experimentally and computationally.^87–89^ Well-known for its disulfide bonding, cysteine is crucial for the structural stability of proteins.^90^ Interestingly, cysteine dimerization is not limited to biological systems or surface interactions; it also occurs rapidly in aqueous solutions. Research shows that about 11% of cysteine molecules form dimers upon dissolving, with complete conversion to cystine occurring within 6 days, highlighting its swift oxidation in water.^91^

From a computational point of view, cysteine dimer formation has been reported during the equilibration phase of some molecular dynamics simulations with ReaxFF^39^ at high zwitterionic cysteine coverage and in the second adsorption layer only. These simulations started from a cysteine droplet far from the surface; therefore, it is not clear whether the dimerization took place in the gas-phase droplet or after reaching the proximity of the surface. In order to gain some further insights into the energetics of dimer formation, we have performed a set of DFT calculations, where we considered both H-bonded and S–S dimers (Scheme 2).

**Scheme 2:**
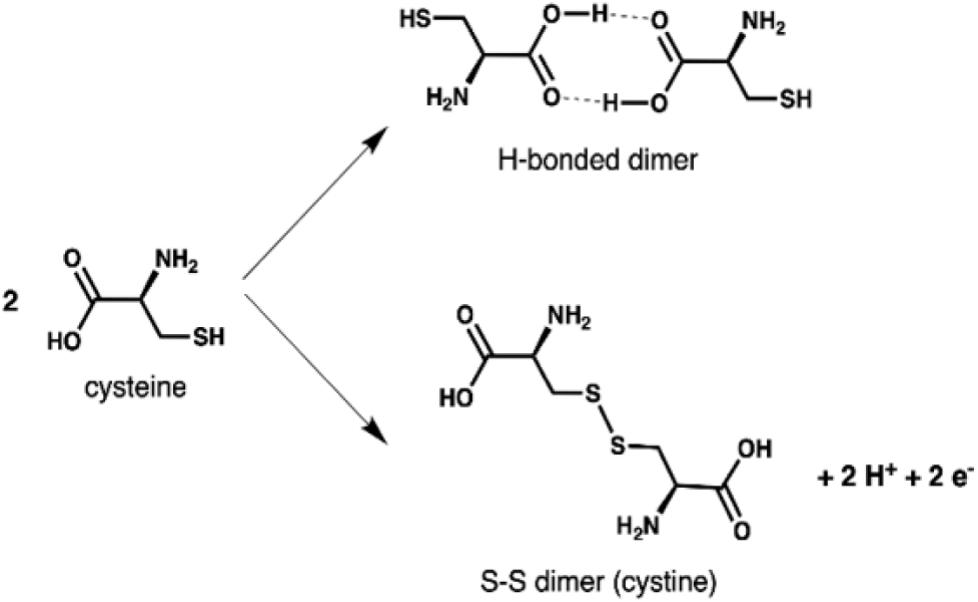
Cysteine H-Bonded (Top) and S–S (Bottom) Dimers Formation

The H-bonded dimers involve the carboxylic acid functionality of two cysteine molecules, and their formation inhibits the deprotonation to carboxylates and the coordination of carboxylate O atoms to surface Ti_5c_ atoms. As a result, H-bonded dimers must establish different types of interactions with the surface, such as amino N atom coordination and thiol S coordination to surface Ti_5c_ atoms. We have evaluated both possibilities, as shown in Figure S6a,b. However, the adsorption energies are far lower than those computed for two adsorbed DP cysteines in close proximity (−3.21 vs −4.86 eV, reported in Figure S4 and Table S3). Therefore, we discard this possibility and conclude that H-bonded dimers cannot be formed on the surface since carboxylic O atoms prefer to form a coordinative bond with surface Ti atoms. The S–S dimers impose some structural constraints, but they do not inhibit adsorption by carboxylated O atoms or by amino N atoms to surface Ti atoms. To support this, we computed the molecular electrostatic potential (MEP) map of the doubly deprotonated S–S dimer (Figure S5), identifying four electron-rich regions corresponding to the two −COO^–^ and two −NH_2_ groups. The carboxylates exhibit a higher electron density due to the extra electrons localized on the oxygen atoms after deprotonation. The disulfide (S–S) bridge shows intermediate character and is not expected to act as a binding site on the polar TiO_2_ surface. Therefore, the −COO^–^ and −NH_2_ groups are the most likely binding sites, interacting with positively charged Ti atoms. These dimers could adsorb lying along the [001] or the [1̅10] directions (in Figure 6). In the latter case, they must bridge over a row of surface O atoms. We investigated both options and obtained adsorption energies that are larger (in the range between −3.87 and −4.21 eV) than for the H-bonded dimers but still quite lower than for two adsorbed DP cysteines in close proximity (−4.86 eV, Figure S4). There is indeed an energy cost to form S–S dimers since this reaction causes oxidation of the cysteine molecules (oxidation state of the S atom goes from −2 to −1). Therefore, the TiO_2_ surface becomes reduced and protonated, as a consequence of the SH dissociation. We also considered the possibility of a molecular H_2_ release instead of surface reduction; however, this results in an even less exothermic process (−3.13 eV). All of the calculated adsorption energy values are shown in Table S7. Additionally, the results of the charge analysis and NCI analysis are presented in Table S8 and Figure S8, respectively.

**Figure 6:**
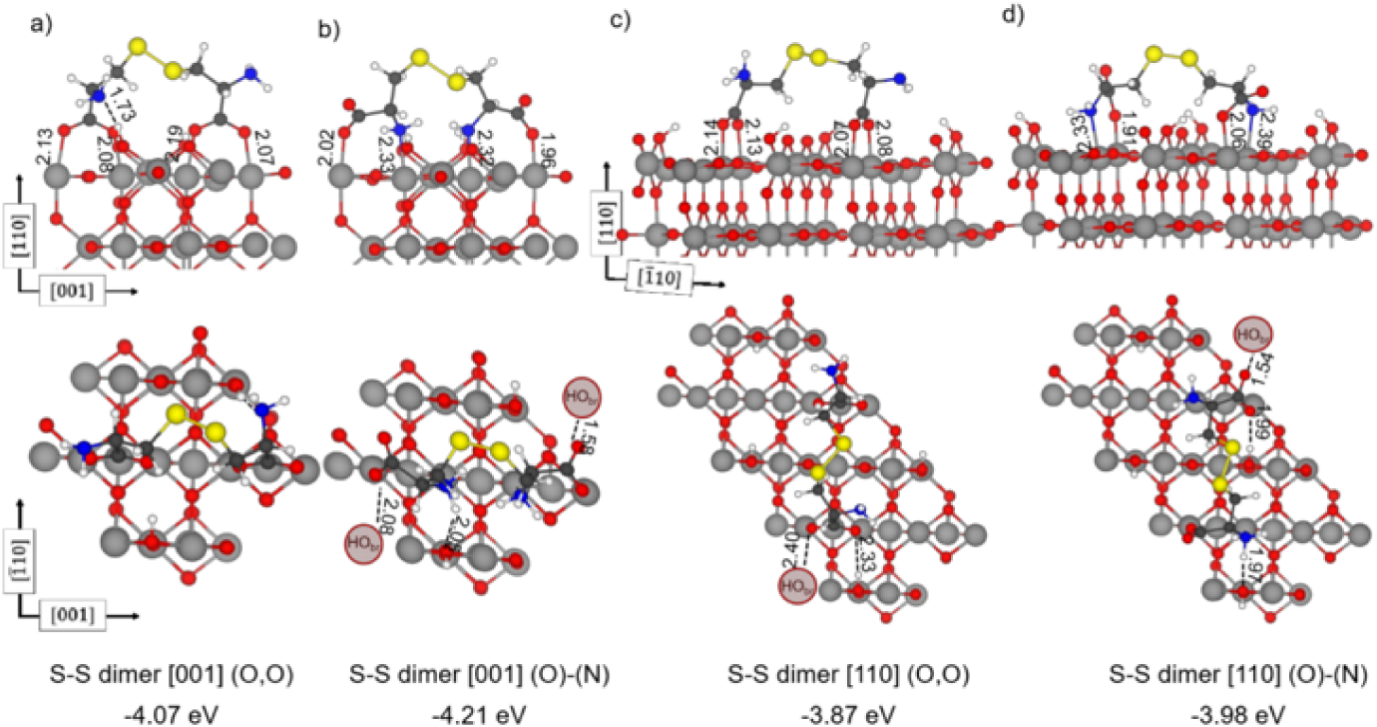
Structures of S–S bonded cysteine dimers adsorbed on the rutile TiO_2_(110) surface along the (a,b) [001] and (c,d) [1̅10] directions. Gray, red, blue, yellow, white, and black spheres represent Ti, O, N, S, H, and C atoms, respectively. Dashed lines indicate hydrogen bonds and other electrostatic interactions. Adsorption energies (in eV) calculated with PBE-D3+U and relevant bond lengths (values in Å) are reported.

Finally, we evaluated the possibility of S–S dimer dissociation on the surface (Figure S7) for the S–S dimer [001] (O)-(N), but this process is found to be largely endothermic by +2.32 eV. Therefore, we conclude that S–S dimers do not form after monomer adsorption on the surface but are either present in the cysteine powder or form in the gas phase. Once they reach the surface, they remain stable and do not dissociate. Cysteine oxidation to cystine has been described in numerous studies, and cystine has been found present in multiple cysteine samples as it spontaneously oxidizes in the presence of O_2_ or water.^91–93^

The theoretical spectroscopic characterization based on CLSs of the adsorbed cysteine S–S dimer [001] (O)-(N) (see Table S4) reveals no significant differences from what we reported above for the deprotonated monomeric configurations DP (O)-(N) and DP (O,O) for all investigated atoms. This is because the S 2p CLSs of the S–S group are very close to those of the SH one. Only minor differences can be noticed for the O 1s CLSs of the H-bonded O atom (O–HO_br_).

## Conclusions

This study presents a comprehensive exploration of cysteine adsorption on the rutile TiO_2_(110) surface through a combined experimental and theoretical approach. Our results indicate that the bidentate (O)-(N) binding configuration is more stable than the previously proposed (O,O) bridging mode, which had been considered the global minimum in previous studies.^36,37^

Additionally, we identified a previously unreported (O)-(S) binding geometry, demonstrating that sulfur can chemisorb without breaking the C–S bond. This finding explains the low binding energy observed by Ataman et al. and challenges earlier assignments that assumed dissociative adsorption.^34^ Our XPS and FT-IRRAS results, supported by solvated DFT models, also confirm that cysteine becomes a zwitterionic form when a minimal hydration shell forms, providing experimental validation for trends previously predicted for amino acids on TiO_2_ surfaces. Finally, STM imaging corroborates our findings by directly visualizing both monomeric and dimeric arrangements, the latter stabilized by intermolecular disulfide bonds, species that had until now only been proposed by ReaxFF simulations.^39^

Overall, our study clarifies how cysteine, a multifunctional amino acid, binds to the rutile TiO_2_(110) surface. These atomistic insights serve as a foundation for understanding the adsorption behavior of larger biomolecules such as peptides and proteins, whose interactions with TiO_2_ surfaces are critical in a variety of technological applications. This knowledge is key for developing surface functionalization strategies that can tailor the TiO_2_ properties for specific applications. In biomedicine, this can guide the design of coatings and implants with predictable biomolecular interactions. In catalysis and environmental technologies, it can help to enhance photocatalytic performance, where surface chemistry and the adsorption of organic molecules play a central role.

## Supplementary Material



## Notes

Crystal preparation methods; cysteine evaporation details; experimental procedures for X-ray photoelectron spectroscopy (XPS); scanning tunneling microscopy (STM); and Fourier-transform infrared reflection absorption spectroscopy (FT-IRRAS); thickness estimation procedure; detailed computational methodology including density functional theory (DFT) calculations, adsorption energy evaluations, and core-level shift calculations; additional figures and tables illustrating surface characterization, calculated binding geometries, adsorption energies, and core-level shifts (PDF)

## Abbrevations

TiO_2_: Titanium dioxide
Cys: cysteine
XPS: X-ray photoelectron spectroscopy
FT-IRRAS: Fourier-transform infrared reflection absorption spectroscopy
STM: scanning tunneling microscopy
DFT: density functional theory
SARS-CoV-2: severe acute respiratory syndrome coronavirus 2
CLS: core-level shift
LEED: low-energy electron diffraction
DP: deprotonated
ZW: zwitterionic
