# Supplementary material for: Unraveling the Role of the Multifunctional Groups in the Adsorption of l‑Cysteine on Rutile TiO2(110)
Source: J Am Chem Soc. 2025 Oct 22;147(44):40158–70. doi: 10.1021/jacs.5c07119 (PMC12593349; doi:10.1021/jacs.5c07119)
Supplement: Supplementary file 1 [file ja5c07119_si_001.pdf]

Supporting Materials for

Unravelling the Role of the Multi-Functional Groups in the Adsorption of L-Cysteine on Rutile TiO_2 (110)

Miguel Blanco Garcia[1,2], Daniele Perilli[3], Chiara Daldossi[3], Aldo Ugolotti[3], Martina Giordano[3], Daniel Silvan Dolling[1,2], Michael Wagstaffe[1], Mona Kohantorabi[1], Andreas Stierle[1,2], Cristiana Di Valentin[3,4*], Heshmat Noei[1*]

AUTHOR ADDRESS

[1] Centre for X-ray and Nano Science CXNS, Deutsches Elektronen-Synchrotron DESY, 22603 Hamburg, Germany

[2] University of Hamburg, Notkestraße 9-11, 22607 Hamburg, Germany

[3] Department of Materials Science, University of Milano-Bicocca, Via R. Cozzi 55, I-20125, Milano, Italy

[4] BioNanoMedicine Center NANOMIB, University of Milano-Bicocca, I-20125, Milano, Italy

* Email: cristiana.divalentin@unimib.it and heshmat.noei@desy.de

S.I. 1 EXPERIMENTAL AND COMPUTATIONAL DETAILS

1.1 Crystal Preparation methods:

The single crystalline rutile TiO_2(110) sample, 8 mm × 8 mm × 2 mm (Surface Net Ltd.), was subjected to multiple cycles of argon ion sputtering (1 keV, 10^{-6} mbar Ar^+, 5 minutes) and subsequent annealing. The annealing process was carried out at 1000 K under back pressure of 10^{-6} mbar of O_2 for 10 minutes, followed by an additional 10 minutes annealing under ultra-high vacuum (UHV) at the same temperature. This careful preparation resulted in a flat and uncontaminated rutile surface with minimal Ti^{3+} defect sites as it was reported by Diebold et al. (*1*). The surface was prepared until a clean, well-defined (1 x 1) low energy electron diffraction (LEED) pattern was achieved, and XPS analysis confirmed the absence of contaminants in O 1s and C 1s core levels, as well as an almost defect free surface shown in the minimal content of Ti^{3+} as shown in **Figure S1.**

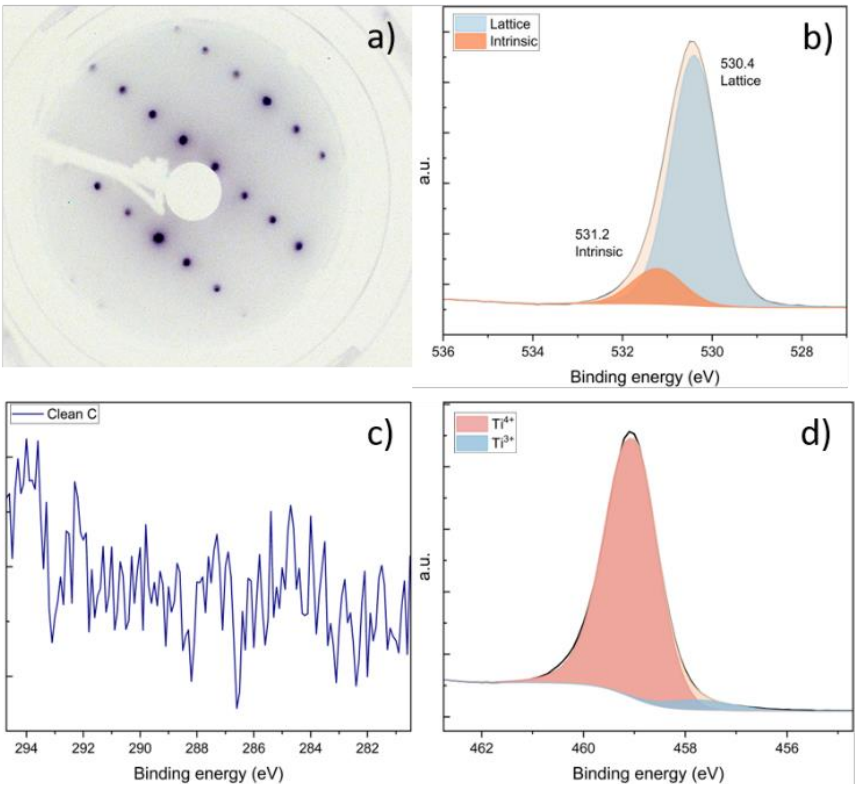

Figure S1. (a) LEED pattern of rutile (110) after preparation under UHV taken at 100 eV. Experimental and deconvoluted XP spectra of the clean rutile (110) surface (b) O 1s, (c) C 1s, (d) Ti 2p 3/2.

1.2 Evaporation of cysteine:

Cysteine evaporation was conducted in two different UHV sample preparation chambers. For XPS and STM measurements, evaporation was done in a load lock connected to the STM chamber with a base pressure of low 10^{-9} mbar. When measuring XPS, the sample had to be transferred after the evaporation through a tunnel that connects all equipment with a base pressure of low 10^{-9} mbar requiring about 15 minutes to do all the transfer process.

The FT-IRRAS chamber has a base pressure of low 10^{-10} mbar and is equipped with evaporation line and a nuzzle, which is directed to the sample so the deposition and the measurement can be done without moving the sample from the measuring stage.

For all experiments the gas line containing cysteine is pumped and baked up and then heated up to 130 °C during exposure. The temperature was measured with a type k thermocouple attached to the glass tube.

1.3 X-ray photoelectron spectroscopy:

All XPS measurements were carried out at the DESY Nanolab at the Centre for X-ray and Nano Science, DESY, Hamburg (2). The instrument is equipped with a monochromated Al Kα source (1.486 keV) and a Phoibos 150 hemispherical energy analyser, operating under a base pressure of low 10^{-10} mbar. Data analysis was performed using CasaXPS software.

1.4 Scanning tunnelling microscopy:

STM images were acquired using a combined STM/AFM system operating under UHV conditions at a pressure of low 10^{-11} mbar (2). Measurements were conducted in constant current mode at room temperature, employing a tungsten tip. Scanning parameters included voltage ranges from 1.4 V to 1.8 V and current ranges from 0.05 nA to 0.3 nA.

1.5 Fourier-Transformed Infrared Spectroscopy:

FT-IRRAS spectrometer (Bruker Vertex V80) is connected to a UHV chamber with a base pressure of low 10^{-10} mbar. Each IR spectrum was taken in reflection mode with 512 scans at a resolution of 2 cm^{-1}.

1.6 Thickness estimation:

The thickness of the cysteine layer (d_{meas}) was estimated by calculating the area intensity of the TiO_2 $2p_{3/2}$ peak using the following formula (3).

$$I_M = I_0 \cdot e^{\frac{-d_{meas}}{\lambda}}$$

In this formula, I_M represents the area of the Ti $2p_{3/2}$ peak in $TiO_2(110)$ after cysteine adsorption, I_0 denotes the area of the same peak for the clean surface, and λ stands for the effective attenuation length (EAL) of electrons in L-cysteine, extracted from the NIST Database 82 (4). The electrons in Ti $2p_{3/2}$ have a kinetic energy of 1027.56 eV and have an asymmetry parameter (β) of 1.41. We consider the stoichiometric coefficients for cysteine: H→7, C→3, N→1, O→2, S→1; it has 42 valence electrons, a band-gap energy of 4.62 eV and a density of 1.68 g/cm^3 (5). The incidence and emission angles of the XPS set up are 55^0 and 0^0, correspondingly.

The average EAL for Ti $2p_{3/2}$ Ek = 1027.56 eV is recorded as EAL = 27.4, providing a practical average EAL value applicable for thicknesses up to 5 nm. I_0 has a value of 56031 counts per seconds cps and I_m has 51354 cps.

The calculated total thickness approximates to 0.3 nm, signifying nearly half a monolayer of cysteine, assuming its dimensions as 6.4 Å in length and 3.7 Å in width (6).

To further understand the surface coverage, we perform a STM study with the rutile (110) surface covered with more than 50 L. **Figure S2** presents a STM image of a rutile (110) surface nearly fully saturated with 50 L of cysteine. At this coverage the whole surface is fully covered, however we do not observe any bi- or multilayer formations as it was also calculated in XPS. This suggests that there is a limit below a monolayer in which cysteine can adsorb on TiO_2 at room temperature. At this coverage, no distinct preferred orientation or ordered molecular layer of cysteine can be discerned on TiO_2 surface, apart from the ordered features highlighted in blue circles. It is possible to observe individual cysteine molecules (red circles) as well as dimers (yellow circles). Blue stripes align preferentially along the [001] direction of the surface and being two cysteine molecules in width. Data from both XPS and STM suggest that the rutile surface becomes saturated with a specific quantity of cysteine at room temperature but a multilayer does not form.

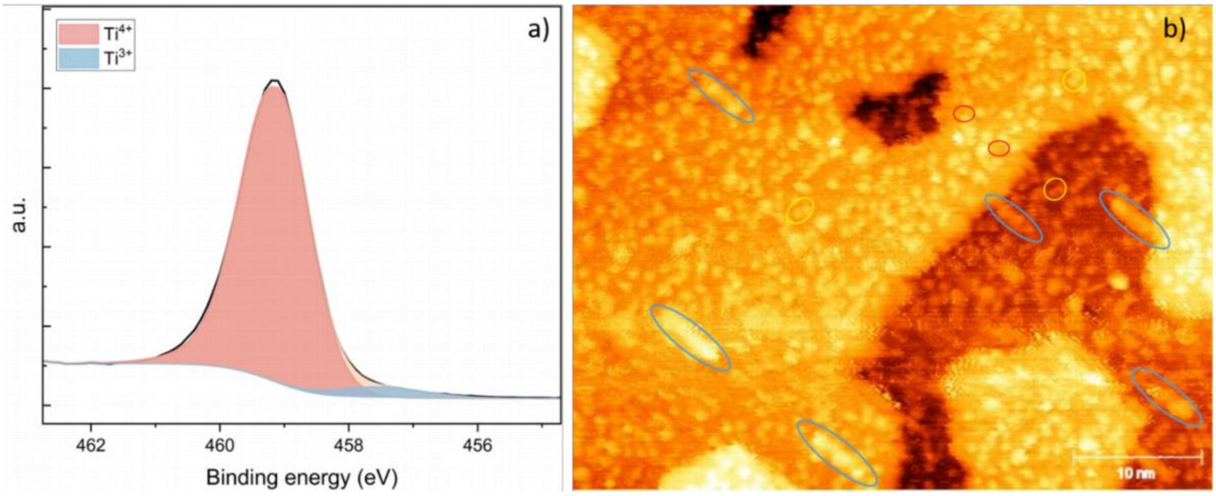

Figure S2. (a) XP spectra of Ti 2p 3/2 region after cysteine evaporation. (b) STM image of rutile (110) surface measured after dosing 50 L of cysteine at room temperature on a pre-cleaned surface. 35x45 nm; image taken with 1.7 V of bias voltage and 0.3 nA current.

1.7 Computational details:

Density Functional Theory (DFT) calculations were performed using the plane-wave-based Quantum ESPRESSO (QE) package (*7, 8*). Ultrasoft pseudopotentials (*9*) were employed to model the electron-ion interactions, with energy cutoffs of 52 Ry for kinetic energy and 575 Ry for charge density expansion, applied in all calculations. The Perdew-Burke-Ernzerhof (PBE) functional (*10*) was used for the electron exchange-correlation, while van der Waals interactions were included using the DFT-D3 correction (*11*). A Hubbard U value of 3 eV was applied to Ti, in accordance with previous literature (*12*).

The rutile $TiO_2(110)$ surface was modelled using a slab of four O-Ti-O trilayers, with the oxygen and titanium atoms in the bottom trilayer (only the bottom oxygen in this layer was allowed to relax) fixed at their bulk positions during geometry optimization to simulate a semi-infinite solid. To vary the number of five-coordinated titanium (Ti_{5c}) rows, two supercell models were employed: a c(4×2) surface cell for the case with one Ti_{5c} row, and a c(8×2) cell for the case with two Ti_{5c} rows. A vacuum space of at least 13 Å was included in the direction perpendicular to the surface to prevent interactions between adjacent periodic images. Ball-and-stick models were visualized using VESTA software (*13*).

Geometry optimizations were carried out starting from a range of initial configurations, generated by manually placing the cysteine molecule on the rutile surface, guided by the expected chemical reactivity of its functional groups, namely $-NH_2$, $-COOH$, and $-SH$, on a polar oxide surface. All geometry optimizations were performed at the Γ point. To verify convergence, additional tests using a denser 4×4×1 k-point mesh were carried out on all configurations presented in Figures 3abc, 4b, and 6ab of the main text, revealing tiny differences in both geometries and energetics.

The adsorption energies (ΔE_{ads}) in vacuum for cysteine on the $TiO_2(110)$ surface were calculated using the following formula:

$$\Delta E_{ads} = E_{n\ cys/TiO2} - (nE_{cys} + E_{TiO2})$$

where $E_{n\ cys/TiO2}$ is the total energy of the optimized system with n adsorbed cysteine molecules (where n = 1 or 2), E_{cys} is the total energy of an isolated cysteine molecule in the gas phase, and E_{TiO2} is the total energy of the optimized $TiO_2(110)$ surface without any adsorbed cysteine molecules. For cases where the adsorption of a single cysteine molecule was computed in the presence of water molecules, the adsorption energies were determined as follows:

$$\Delta E_{ads} = E_{cys+nH2O/TiO2} - (E_{cys} + nE_{H2O} + E_{TiO2})$$

where $E_{cys+nH2O/TiO2}$ is the total energy of the system with a cysteine molecule (in its deprotonated or zwitterionic form) adsorbed on the TiO_2 surface in the presence of n water molecules, and E_{H2O} is the total energy of a water molecule in the gas phase.

In the case of cystine formation, where a disulphide (S-S) bond is formed between two cysteine molecules, we also consider the possibility of the formation of an H_2 molecule in the gas phase. In this case, the adsorption energy for cystine on the $TiO_2(110)$ surface is calculated using the following equation:

$$\Delta E_{ads,cystine} = E_{cystine/TiO2} + E_{H2} - (2E_{cys} + E_{TiO2})$$

where $E_{cystine/TiO2}$ is the total energy of the optimized system with cystine adsorbed on the TiO_2 surface, E_{H2} is the total energy of the H_2 molecule in the gas phase, E_{cys} is the energy of an isolated cysteine molecule in the gas phase, and E_{TiO2} is the energy of the TiO_2(110) surface without any adsorbed molecules.

XPS calculations were performed within the ΔSCF method (*14*) with the use of additional pseudopotentials for each inequivalent C, N, O or S atom, generated removing an electron from the core 1s (C, N, O) or 2p (S) orbitals. For these simulations, the plane-waves basis-set needed to be enlarged to 74 and 575 Ry. Such an approach does not allow the calculation of an absolute value of the binding energies (BEs), but just their difference with respect to a chosen reference, or core level shift (CLS). To be able to compare the CLSs of different structures contained in distinct simulation cells, we placed one CH_4, N_2, O_2 or H_2S reference molecule at a time for any C, N, O and S photoexcited atom, respectively. In order to minimize the interaction of these molecules with the simulated system, we inserted them in the vacuum region, with a distance of at least 12 Å from any other atom and enlarged the supercell size accordingly.

The vibrational frequencies of cysteine adsorbed on TiO_2(110) were calculated within the harmonic approximation. Numerical Hessian matrices were constructed by applying finite displacements (±0.02 a.u.) along all three Cartesian directions from the equilibrium positions and computing the corresponding atomic force components. This procedure was applied to both the gas-phase and adsorbed configurations. The resulting Hessian matrices were diagonalized at the Γ point to obtain the vibrational frequencies associated with each normal mode. To decouple the motion of relaxed atoms from those constrained to their bulk positions, the Hessian matrix elements involving atoms constrained to simulate the bulk were set to zero.

Thanks to the computed vibrational frequencies, the free energies (G) of cysteine adsorbed on TiO_2(110) at 298.15 K were obtained by correcting the electronic energies (E) for zero-point energy (ZPE), thermal contributions to the enthalpy (∫CpdT), and the vibrational entropy (S_{vib}), according to the following expression:

$$G = E + ZPE + \int C_p dT - TS_{vib}$$

For the gas-phase cysteine molecule, the ideal gas approximation was applied, assuming a standard pressure of 1 bar.

Finally, atomic charge analysis was performed using the Hirshfeld method (*15*) and non-covalent interaction (NCI) analysis was carried out using the Critic2 code (*16*)

S.I. 2 Calculated binding geometries and energies for DP-Cysteine on rutile (110) at low and high coverage

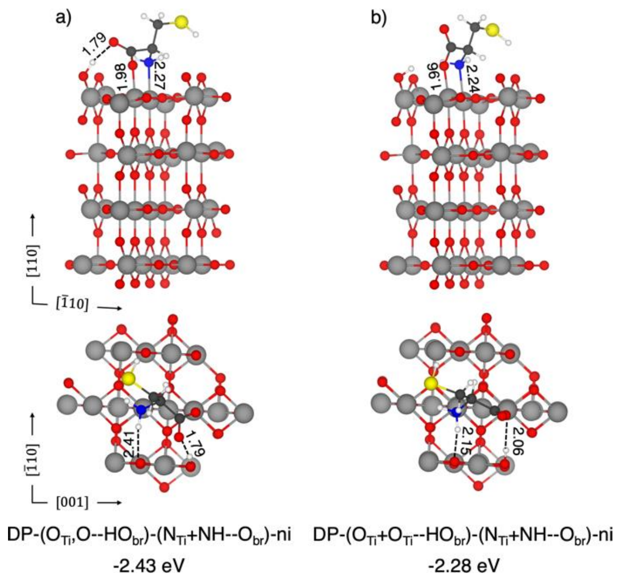

Figure S3. Structures of deprotonated cysteine adsorbed on rutile TiO_2 (110) surface. (a) The newly optimized DP-(O_{Ti},O--HO_{br})-(N_{Ti},NH--O_{br})-ni with a H-bond between the HO_{br} and the unbound carboxylate O atom, and (b) DP-(O_{Ti}+O_{Ti}--HO_{br})-(N_{Ti}+NH--O_{br})-ni, based on the structure calculated by Muir and Idriss with a H-bond between the HO_{br} and the Ti-bound carboxylate O atom. Gray, red, blue, yellow, white, and black spheres represent Ti, O, N, S, H, and C atoms, respectively. Dashed lines indicate hydrogen bonds and other electrostatic interactions. Adsorption energies (in eV) calculated with PBE+U and relevant bond length (values in Å) are reported.

Table S1. Adsorption energy values (in eV) calculated using PBE without and with Hubbard U correction for cysteine molecules adsorbed on rutile TiO_2(110) surface.

ΔE_{ads} (eV)	Figure	PBE-D3	PBE-D3+U
DP-(O_{Ti},O_{Ti})-ni-(HS--HO_{br})	1a	-1.90	-2.08
DP-(O_{Ti}+O_{Ti}--HO_{br})-(N_{Ti}+NH--O_{br})-ni	S3b	-2.08	-2.28
DP-(O_{Ti},O--HO_{br})-(N_{Ti}+NH--O_{br})-ni	1b	-2.26	-2.43
biDP-(O_{Ti},O--HO_{br})-ni-(S_{Ti})	1c	-1.79	-2.04
DP-(O_{Ti})-ni-(HS_{Ti})	1d	-1.06	-1.19
ZW-(O_{Ti},O_{Ti})-ni-(SH--O_{br})	4a	-1.75	not found

Table S2. Adsorption energy values (in eV) calculated using PBE with and without Grimme D3 dispersion correction for cysteine molecules adsorbed on rutile TiO_2(110) surface, compared with those obtained from in a previous work.

ΔE_{ads} (eV)	PBE-D3	PBE	PBE (Muir and Idriss)
DP-(O_{Ti},O_{Ti})-ni-(HS--HO_{br})	-1.90	-1.38	-1.47
DP-(O_{Ti}+O_{Ti}--HO_{br})-(N_{Ti}+NH--O_{br})-ni	-2.08	-1.33	-1.39

This result is in contrast with what reported in a previous study Muir and Idriss (*17*) where the (O,O) was indicated as the best adsorption mode. This is due to two reasons: 1) in that work a different (O)-(N) structure was proposed (DP-(O_{Ti}+O_{Ti}--HO_{br})-(N_{Ti}+NH--O_{br})-ni in **Figure S3b**) than the one found in the present work (DP-(O_{Ti},O--HO_{br})-(N_{Ti}+NH--O_{br})-ni in **Figure 1b**), which is more stable by -0.15 eV. The difference is related to the O atom involved in the H-bond. 2) The second reason is the lack of dispersion interactions in the DFT study by Muir and Idriss as we proved with some additional calculations without the Grimme correction in **Table S2**.

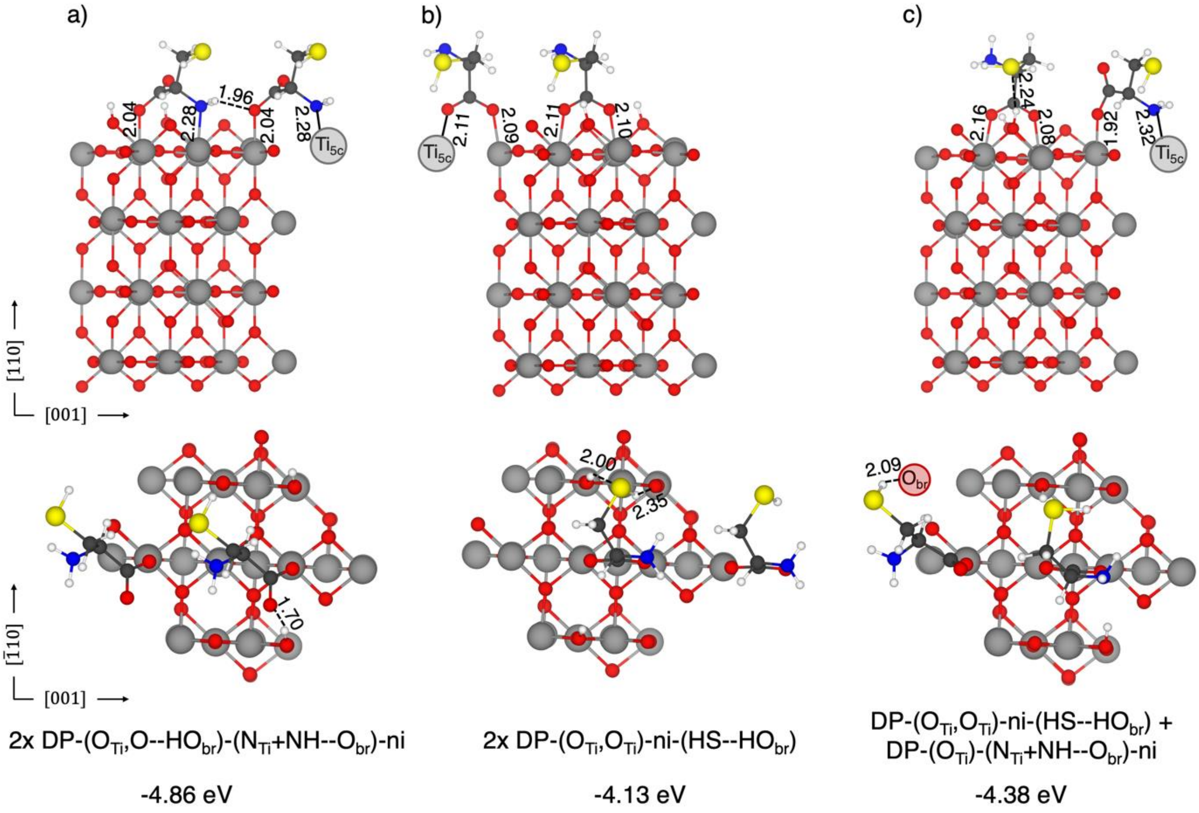

Figure S4. High coverage cysteine adsorption on rutile TiO_2(110) surface. (a) Two cysteine molecules in the DP-(O_{Ti},O--HO_{br})-(N_{Ti}+NH--O_{br})-ni configuration, (b) two in the DP-(O_{Ti},O_{Ti})-ni-(HS--HO_{br}) configuration, and (c) one in the DP-(O_{Ti})-(N_{Ti}+NH–O_{br})-ni configuration with one in the DP-(O_{Ti},O_{Ti})-ni-(HS--HO_{br}) configuration are adsorbed on the rutile TiO_2(110) supercell

slab model. Gray, red, blue, yellow, white, and black spheres represent Ti, O, N, S, H, and C atoms, respectively. Adsorption energies (in eV) calculated with PBE-D3+U and relevant bond length (values in Å) are reported.

Table S3. Adsorption energy values (in eV) calculated using PBE-D3 without and with Hubbard U correction for cysteine molecules adsorbed on rutile TiO_2(110) surface at high coverage.

	PBE-D3		PBE-D3+U	
	ΔE_{ads} (eV)	ΔE_{ads} (eV/cys)	ΔE_{ads} (eV)	ΔE_{ads} (eV/cys)
2x DP-(O_{Ti},O--HO_{br})-(N_{Ti}+NH--O_{br})-ni	-4.52	-2.26	-4.86	-2.43
2x DP-(O_{Ti},O_{Ti})-ni-(HS--HO_{br})	-3.79	-1.89	-4.13	-2.06
DP-(O_{Ti},O_{Ti})-ni-(HS--HO_{br}) + DP-(O_{Ti})-(N_{Ti}+NH--O_{br})-ni	-4.03	-2.01	-4.38	-2.19

S.I. 3 Core Level shifts and XP fitting information

3.1 Core level shifts information:

We focused on the DP-(O_{Ti},O--HO_{br})-(N_{Ti}+NH--O_{br})-ni, DP-(O_{Ti},O_{Ti})-ni-(HS--HO_{br}) and biDP-(O_{Ti},O--HO_{br})-(ni)-(S_{Ti}) configurations, which are prototypes of the different geometries a cysteine monomer can adsorb on the rutile TiO_2(110) surface: DP (O)-(N), DP (O,O), DP (O)-(S). For the first structure, we considered also the corresponding high coverage configuration (2x DP(O)-(N)).

Table S4. CLSs (in eV) calculated for selected adsorption configurations, the isolated molecule and the rutile TiO_2(110) surface (for the topmost layer only) for different photoexcited core electron, each with respect to its reference.

*: deprotonated OH.

**: we obtain the same CLS for the two molecules included in the supercell.

Èdge	Atom type	DP (O)-(N)	DP (O,O)	DP (O)-(S)	2x DP (O)-(N)**	ZW (O,O) + H_2O	S-S dimer [001] (O)-(N)	Cys gas phase	TiO_2 surface
C 1s	C-O	+2.8	+2.8	+2.8	+2.7	+2.9	+2.3/+2.2	+4.0	-
	C-N	+0.7	+0.8	+0.3	+0.6	+1.9	+0.7/+0.8	+1.4	-
	C-S	+0.3	+0.3	-0.5	+0.2	+0.7	+0.2/+0.0	+0.6	-
N 1s	NH_2	-	-1.7	-2.0	-	-	-	-2.2	-
	NH_2-Ti_{5c}	-1.0	-	-	-1.3	-	-0.1/-0.3	-	-
	NH_3^+	-	-	-	-	+1.6	-		
O 1s	O1-Ti	-1.1	-0.5	-0.9	-1.1	-0.2	-0.6/-0.8	-	-
	O2-Ti	-	-0.6	-	-	-0.4	-	-	-
	O2*	-1.2	-	-0.9	-1.4	-	-0.9/-1.1	-	-
	C-OH/C=O	-	-	-	-	-	-	+0.9/-1.1	-
	TiO_2 O_{br}	-2.7	-2.7	-2.4	-2.5	-2.9	-0.6	-	-3.0
	TiO_2 O_{br}1H	-1.5	-1.4	-1.1/-1.6	-1.4	-	-0.6	-	-
	TiO_2 O_{3c}	-2.3	-2.3	-2.1	-2.3	-2.3	-1.9	-	-2.4
S 2p	S-H	-0.7	-0.2	-	-0.7	-1.1	-	-0.8	-
	S-Ti	-	-	-2.5	-	-	-	-	-
	S-S	-	-	-	-	-	-0.5/-0.4		

3.2 Peak fitting protocol:

For each core level, deconvolution started with the minimum set of chemically plausible components; an additional peak was introduced only when demanded by either the envelope shape or the residual trace.

C 1s: L-cysteine has three inequivalent carbon atoms (C-SH, C-NH_2, COOH), so three components were fitted. A fourth peak did not improve the residual and was discarded.

O 1s: On clean TiO_2(110), the line consists of a lattice-oxygen peak plus a shoulder from intrinsic defects. Adsorption leaves extra intensity at high binding energy. Adding one component assigns this to carboxylate oxygens and removes the mismatch.

S 2p: The S 2p level is a doublet ($2p_{3/2}$–$2p_{1/2}$, 1.2 eV split, 2 : 1 area). A single -SH doublet left a low BE shoulder. Introducing a second doublet assigned to surface bound thiolate (S-Ti) eliminates the shoulder and flattens the residual.

N 1s: A one peak fit produced an overly broad, asymmetric envelope. Splitting into neutral –NH_2 and protonated –NH_3^+ components removed the asymmetry and gave a satisfactory residual.

Residual standard deviations are compiled in Table S5 and confirm that no further peaks are required.

Table S5. Results of the fitting for XPS data All peaks have been fitted using a Shirley background and a Asymmetric Lorentzian lineshape LA(30) that has been proofed adequate for lightly conductive oxides (*18–20*).

Edge	Surface	Atom type	Position (eV)	FWHM (eV)	Ratio	Residual STD
O 1s	Clean	Intrinsic	531.2	1.31	0.15	1.78
		Lattice	530.4	1.23	1	
	Adsorption	Intrinsic	531.2	1.29	0.17	
		Carboxylate	532.1	1.45	0.06	
Ti 2p	Clean	Ti4+	549.1	1.12	1	3.24
		Ti3+	457.8	1.59	0.5	
	Adsorption	Ti4+	459.1	1.15	1	
		Ti3+	457.6	1.9	0.05	
C 1s	Adsorption	C-O	289.1	1.38	1	0.97
		C-N	286.1	1.69	1.1	
		C-S	285.2	1.47	1.1	
S $2p_{3/2}$	Adsorption	S-H	164.1	1.37	1	0.82
		S-Ti	161.7	1.8	0.27	
N 1s	Adsorption	NH2	400.1	1.5	1	0.52
		NH3+	401.5	1.5	0.6	

S.I. 4 Thermochemical Data

Table S6: Electronic and Gibbs free energies for cysteine adsorption on rutile. The corresponding desorption temperatures, estimated using the Redhead approximation, are reported in the last column.

	**ΔE (eV)**	**ΔG (eV)**	**T_{des} (°C)**
DP-(O_{Ti},O--HO_{br})-(N_{Ti}+NH--O_{br})-ni	-2.43	-1.64	246
DP-(O_{Ti},O_{Ti})-ni-(HS--HO_{br})	-2.08	-1.37	183
biDP-(O_{Ti},O--HO_{br})-ni-(S_{Ti})	-2.04	-1.22	146

S.I. 5 Calculated geometries and energies for cysteine dimers on Rutile (110)

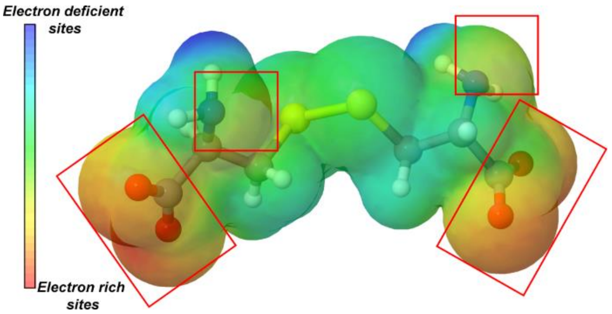

Figure S5. Molecular electrostatic potential (MEP) map of the gas-phase double deprotonated cystine. The MEP is plotted on a 0.0005 a.u. electron density isosurface. Electron-rich regions are highlighted with red boxes.

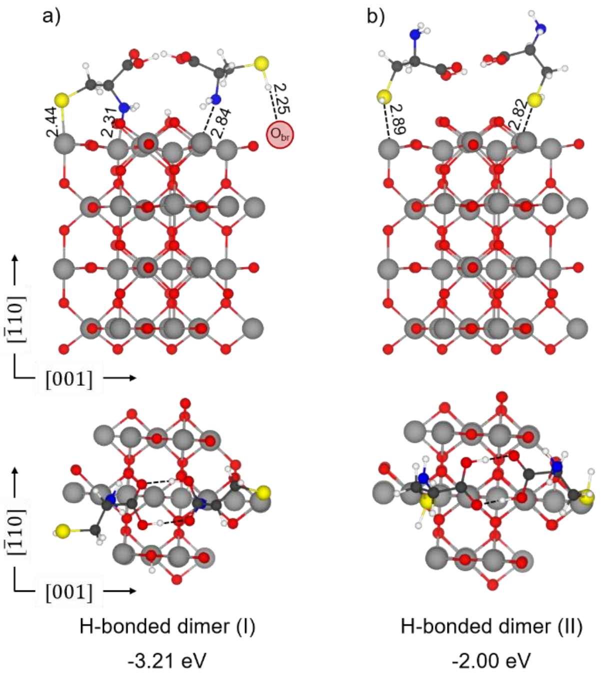

Figure S6. Structures of H-bonded cysteine dimers adsorbed on rutile TiO_2(110) surface by (a) amino N atom coordination and (b) thiol S coordination to surface Ti_{5c} atoms. Gray, red, blue, yellow, white, and black spheres represent Ti, O, N, S, H, and C atoms, respectively. Dashed lines indicate hydrogen bonds and other electrostatic interactions. Adsorption energies (in eV) calculated with PBE-D3+U and relevant bonds length (values in Å) are reported.

Table S7: Adsorption energy values (in eV) calculated using PBE-D3 and PBE-D3+U for cysteine dimers adsorbed on rutile TiO_2 (110). The corresponding structures are shown in **Figure 6** and **S6**.

E_{ads} (eV)	Figure	PBE-D3	PBE-D3+U
H-bonded dimer (I)	S6a	-3.00	-3.21
H-bond dimer (II)	S6b	-1.97	-2.00
S-S dimer [001] (O,O)	6a	-3.40	-4.07
S-S dimer [001] (O)-(N)	6b	-3.58	-4.21
S-S dimer [110] (O,O)	6c	-3.28	-3.87
S-S dimer [110] (O)-(N)	6d	-3.39	-3.98

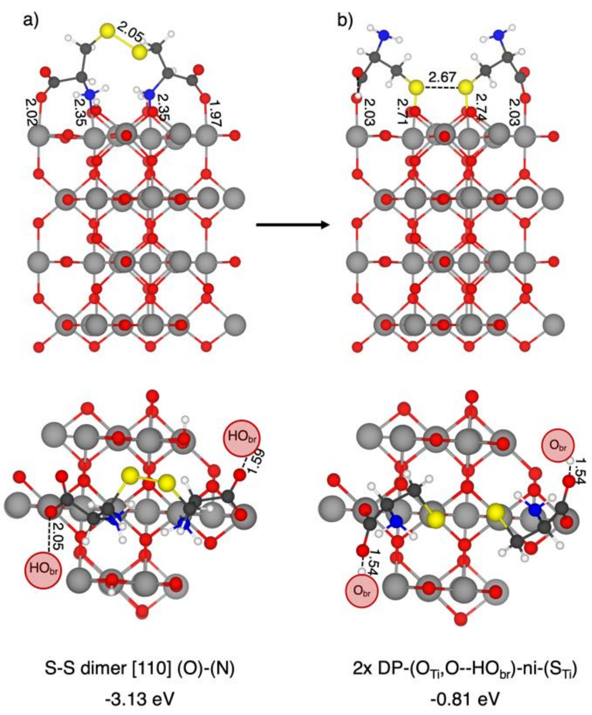

Figure S7. Structures of S-S cysteine dimers dissociation on rutile $TiO_2(110)$ surface. (a) S-S dimer adsorbed on the rutile $TiO_2(110)$ surface. (b) Cysteine monomers, obtained from S-S bond breaking of the dimer, absorbed on the rutile $TiO_2(110)$ surface. Gray, red, blue, yellow, white, and black spheres represent Ti, O, N, S, H, and C atoms, respectively. Dashed lines indicate hydrogen bonds and other electrostatic interactions. Adsorption energies (in eV) calculated with PBE-D3+U and relevant bonds length (values in Å) are reported.

S.I. 6 Charge and non-covalent interaction (NCI) analysis

Table S8**:** Atomic charges (in e) calculated using the Hirshfeld method for the most representative configurations discussed in the main text, along with gas-phase cysteine for comparison.

	**Atomic charge (e)**			
	**O (-/Ti)**	**O (H/Ti)**	**N**	**S**
**Cys**	-0.24	-0.15	-0.21	-0.07
**DP-(O)-(N)**	-0.20	-0.20	-0.10	-0.04
**DP-(O,O)**	-0.15	-0.16	-0.20	-0.01
**DP-(O)-(S)**	-0.16	-0.19	-0.20	-0.09
**ZW-(O,O)**	-0.15	-0.16	-0.02	-0.10
**S-S dimer**	-0.16/-0.16	-0.16/-0.17	-0.12/-0.20	-0.02/+0.01

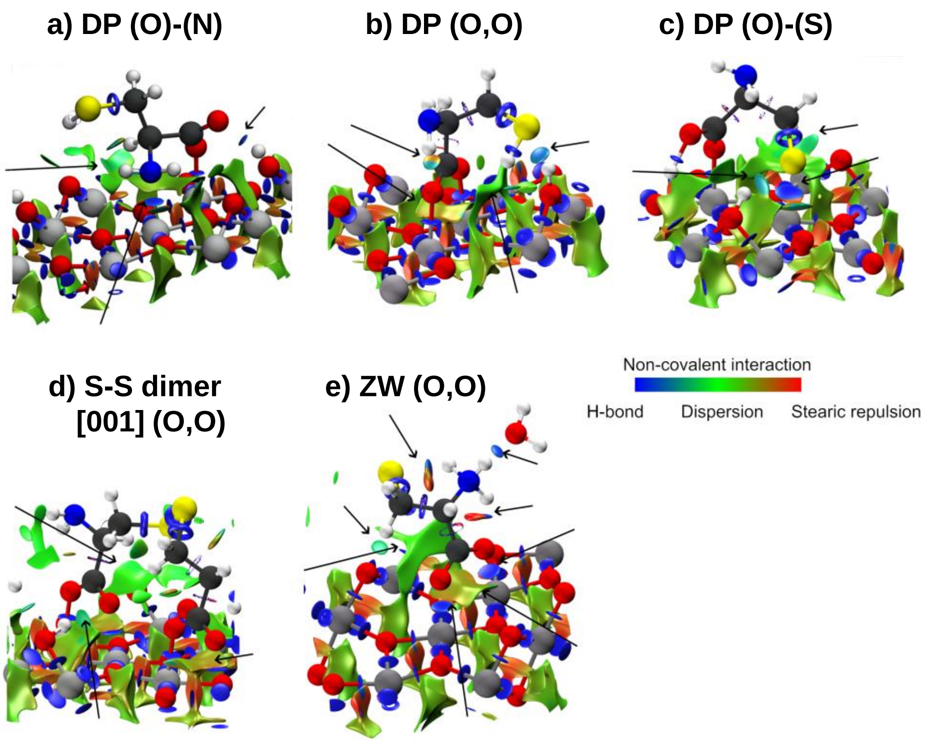

Figure S8. Non-covalent interaction (NCI) isosurfaces for representative configurations of the deprotonated, S–S dimer, and zwitterionic cases, as described in the main text. The color scale distinguishes the types of interactions: H bonding (blue), dispersion forces (green), and steric repulsion (red). Key interactions are highlighted with black arrows in each panel.

S.I. 7 STM population analysis

All high-quality STM images obtained under identical preparation and imaging conditions. Adsorbates were classified by visual inspection as either isolated molecules (red) or paired features consistent with cysteine dimers (yellow). Across the full image set we find an approximate **1 : 4 ratio of dimers to monomers**.

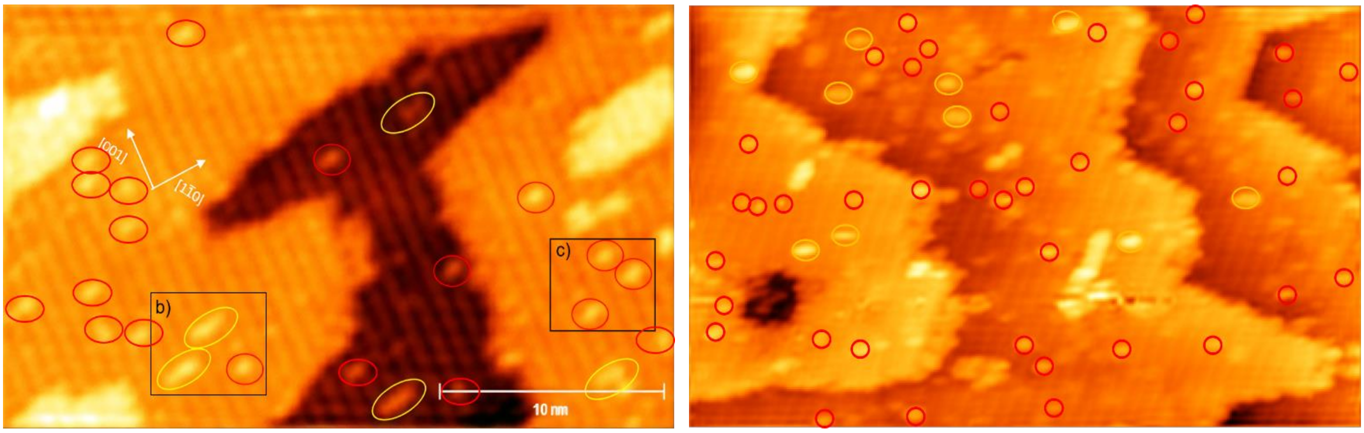

Figure S9. STM high resolution images of 0.1 L of cysteine at room temperature. left) is the image included in the main text with all individual adsorbates counted; right) different region of the sample from the same set.

S.I. 8 FT-IRRAS Background

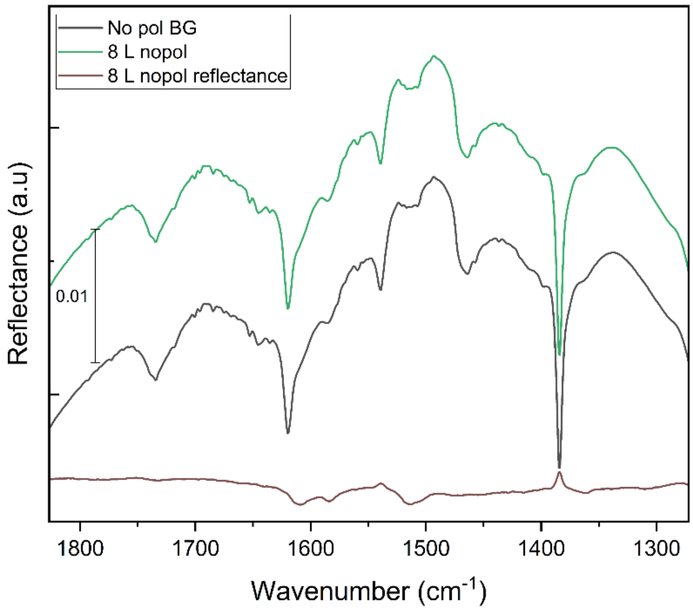

Figure S10. FT-IRRAS absolute data. In black is background used as reference, in green the spectra right after adsorption of 8L of cysteine and in brown the reflectance measured as $\Delta R/R_0$.

References

1. Diebold, U.; Lehman, J.; Mahmoud, T.; Kuhn, M.; Leonardelli, G.; Hebenstreit, W.; Schmid, M.; Varga, P. Intrinsic defects on a TiO2(110)(1×1) surface and their reaction with oxygen: a scanning tunneling microscopy study. *Surface Science* **1998,** *411* (1-2), 137–153. DOI: 10.1016/S0039-6028(98)00356-2.

2. Stierle, A.; Keller, T. F.; Noei, H.; Vonk, V.; Roehlsberger, R. DESY NanoLab. *JLSRF* **2016,** *2*, A76. DOI: 10.17815/jlsrf-2-140.

3. Jablonski, A.; Zemek, J. Overlayer thickness determination by XPS using the multiline approach. *Surface & Interface Analysis* **2009,** *41* (3), 193–204. DOI: 10.1002/sia.3005.

4. Powell, C. J.; Jablonski, A. The NIST Electron Effective-Attenuation-Length Database. *JSA* **2002,** *9* (3), 322–325. DOI: 10.1384/jsa.9.322.

5. Jürgensen, A.; Raschke, H.; Esser, N.; Hergenröder, R. An in situ XPS study of L-cysteine co-adsorbed with water on polycrystalline copper and gold. *Applied Surface Science* **2018,** *435*, 870–879. DOI: 10.1016/j.apsusc.2017.11.150.

6. Ching, C. B.; Hidajat, K.; Uddin, M. S. Evaluation of Equilibrium and Kinetic Parameters of Smaller Molecular Size Amino Acids on KX Zeolite Crystals via Liquid Chromatographic Techniques: Amino acid sizes. *Separation Science and Technology* **1989,** *24* (7-8), 581–597. DOI: 10.1080/01496398908049793.

7. Giannozzi, P.; Baroni, S.; Bonini, N.; Calandra, M.; Car, R.; Cavazzoni, C.; Ceresoli, D.; Chiarotti, G. L.; Cococcioni, M.; Dabo, I.; Dal Corso, A.; Gironcoli, S. de; Fabris, S.; Fratesi, G.; Gebauer, R.; Gerstmann, U.; Gougoussis, C.; Kokalj, A.; Lazzeri, M.; Martin-Samos, L.; Marzari, N.; Mauri, F.; Mazzarello, R.; Paolini, S.; Pasquarello, A.; Paulatto, L.; Sbraccia, C.; Scandolo, S.; Sclauzero, G.; Seitsonen, A. P.; Smogunov, A.; Umari, P.; Wentzcovitch, R. M. QUANTUM ESPRESSO: a modular

and open-source software project for quantum simulations of materials. *J. Phys.: Condens. Matter* **2009,** *21* (39), 395502. DOI: 10.1088/0953-8984/21/39/395502}.

8. Giannozzi, P.; Andreussi, O.; Brumme, T.; Bunau, O.; Buongiorno Nardelli, M.; Calandra, M.; Car, R.; Cavazzoni, C.; Ceresoli, D.; Cococcioni, M.; Colonna, N.; Carnimeo, I.; Dal Corso, A.; Gironcoli, S. de; Delugas, P.; DiStasio, R. A.; Ferretti, A.; Floris, A.; Fratesi, G.; Fugallo, G.; Gebauer, R.; Gerstmann, U.; Giustino, F.; Gorni, T.; Jia, J.; Kawamura, M.; Ko, H.-Y.; Kokalj, A.; Küçükbenli, E.; Lazzeri, M.; Marsili, M.; Marzari, N.; Mauri, F.; Nguyen, N. L.; Nguyen, H.-V.; Otero-de-la-Roza, A.; Paulatto, L.; Poncé, S.; Rocca, D.; Sabatini, R.; Santra, B.; Schlipf, M.; Seitsonen, A. P.; Smogunov, A.; Timrov, I.; Thonhauser, T.; Umari, P.; Vast, N.; Wu, X.; Baroni, S. Advanced capabilities for materials modelling with Quantum ESPRESSO. *J. Phys.: Condens. Matter* **2017,** *29* (46), 465901. DOI: 10.1088/1361-648X/aa8f79}.
9. Dal Corso, A. Pseudopotentials periodic table: From H to Pu. *Computational Materials Science* **2014,** *95*, 337–350. DOI: 10.1016/j.commatsci.2014.07.043.
10. Perdew, J. P.; Burke, K.; Ernzerhof, M. Generalized Gradient Approximation Made Simple. *Physical review letters* **1996,** *77* (18), 3865–3868. DOI: 10.1103/PhysRevLett.77.3865}.
11. Grimme, S.; Antony, J.; Ehrlich, S.; Krieg, H. A consistent and accurate ab initio parametrization of density functional dispersion correction (DFT-D) for the 94 elements H-Pu. *The Journal of Chemical Physics* **2010,** *132* (15), 154104. DOI: 10.1063/1.3382344.
12. Hu, Z.; Metiu, H. Choice of U for DFT+ U Calculations for Titanium Oxides. *J. Phys. Chem. C* **2011,** *115* (13), 5841–5845. DOI: 10.1021/jp111350u.
13. Momma, K.; Izumi, F. VESTA 3 for three-dimensional visualization of crystal, volumetric and morphology data. *J Appl Crystallogr* **2011,** *44* (6), 1272–1276. DOI: 10.1107/S0021889811038970.
14. García-Gil, S.; García, A.; Ordejón, P. Calculation of core level shifts within DFT using pseudopotentials and localized basis sets. *Eur. Phys. J. B* **2012,** *85* (7). DOI: 10.1140/epjb/e2012-30334-5.
15. Hirshfeld, F. L. Bonded-atom fragments for describing molecular charge densities. *Theoret. Chim. Acta* **1977,** *44* (2), 129–138. DOI: 10.1007/BF00549096.
16. Otero-de-la-Roza, A.; Johnson, E. R.; Luaña, V. Critic2: A program for real-space analysis of quantum chemical interactions in solids. *Computer Physics Communications* **2014,** *185* (3), 1007–1018. DOI: 10.1016/j.cpc.2013.10.026.
17. Muir, J.; Idriss, H. Computational study of cysteine interaction with the rutile TiO2 (110) surface. *Surface Science* **2013,** *617*, 60–67. DOI: 10.1016/j.susc.2013.07.009.
18. Engelhard, M. H.; Baer, D. R.; Herrera-Gomez, A.; Sherwood, P. M. A. Introductory guide to backgrounds in XPS spectra and their impact on determining peak intensities. *Journal of Vacuum Science & Technology A: Vacuum, Surfaces, and Films* **2020,** *38* (6). DOI: 10.1116/6.0000359.
19. Major, G. H.; Fairley, N.; Sherwood, P. M. A.; Linford, M. R.; Terry, J.; Fernandez, V.; Artyushkova, K. Practical guide for curve fitting in x-ray photoelectron spectroscopy. *Journal of Vacuum Science & Technology A* **2020,** *38* (6). DOI: 10.1116/6.0000377.
20. Shard, A. G. Practical guides for x-ray photoelectron spectroscopy: Quantitative XPS. *Journal of Vacuum Science & Technology A* **2020,** *38* (4). DOI: 10.1116/1.5141395.
